# Unraveling the cryptic functions of mitogen-activated protein kinases Cpk2 and Mpk2 in *Cryptococcus neoformans*

**DOI:** 10.1128/mbio.01156-24

**Published:** 2024-06-14

**Authors:** Yu-Byeong ­Jang, Jin-Young Kim, Yong-Sun Bahn

**Affiliations:** 1 Department of Biotechnology, College of Life Science and Biotechnology, Yonsei University, Seoul, South Korea; 2 Division of Life Science, College of Life Science and Biotechnology, Yonsei University, Seoul, South Korea; Texas Christian University, Fort Worth, Texas, USA

**Keywords:** Cpk1, Cpk2, Mpk1, Mpk2, MAPK, mating, cell wall integrity

## Abstract

Mitogen-activated protein kinase (MAPK) pathways are fundamental to the regulation of biological processes in eukaryotic organisms. The basidiomycete *Cryptococcus neoformans*, known for causing fungal meningitis worldwide, possesses five MAPKs. Among these, Cpk1, Hog1, and Mpk1 have established roles in sexual reproduction, stress responses, and cell wall integrity. However, the roles of Cpk2 and Mpk2 are less understood. Our study elucidates the functional interplay between the Cpk1/Cpk2 and Mpk1/Mpk2 MAPK pathways in *C. neoformans*. We discovered that *CPK2* overexpression compensates for *cpk1*Δ mating deficiencies via the Mat2 transcription factor, revealing functional redundancy between Cpk1 and Cpk2. We also found that Mpk2 is phosphorylated in response to cell wall stress, a process regulated by the MAPK kinase (MAP2K) Mkk2 and MAP2K kinases (MAP3Ks) Ssk2 and Ste11. Overexpression of *MPK2* partially restores cell wall integrity in *mpk1*Δ by influencing key cell wall components, such as chitin and the polysaccharide capsule. Contrarily, *MPK2* overexpression cannot restore thermotolerance and cell membrane integrity in *mpk1*Δ. These results suggest that Mpk1 and Mpk2 have redundant and opposing roles in the cellular response to cell wall and membrane stresses. Most notably, the dual deletion of *MPK1* and *MPK2* restores wild-type mating efficiency in *cpk1*Δ mutants via upregulation of the mating-regulating transcription factors *MAT2* and *ZNF2*, suggesting that the Mpk1 and Mpk2 cooperate to negatively regulate the pheromone-responsive Cpk1 MAPK pathway. Our research collectively underscores a sophisticated regulatory network of cryptococcal MAPK signaling pathways that intricately govern sexual reproduction and cell wall integrity, thereby controlling fungal development and pathogenicity.

**IMPORTANCE:**

In the realm of fungal biology, our study on *Cryptococcus neoformans* offers pivotal insights into the roles of specific proteins called mitogen-activated protein kinases (MAPKs). Here, we discovered the cryptic functions of Cpk2 and Mpk2, two MAPKs previously overshadowed by their dominant counterparts Cpk1 and Mpk1, respectively. Our findings reveal that these “underdog” proteins are not just backup players; they play crucial roles in vital processes like mating and cell wall maintenance in *C. neoformans*. Their ability to step in and compensate when their dominant counterparts are absent showcases the adaptability of *C. neoformans*. This newfound understanding not only enriches our knowledge of fungal MAPK mechanisms but also underscores the intricate balance and interplay of proteins in ensuring the organism’s survival and adaptability.

## INTRODUCTION

Mitogen-activated protein kinases (MAPKs) are Ser/Thr kinases with crucial roles in growth, stress response, mitosis, apoptosis, differentiation, and survival in eukaryotic organisms. Three evolutionarily conserved tiers of kinases, activated successively by phosphorylation, make up the MAPK pathway: MAPK, MAP2K, and MAP3K (1). The dual phosphorylation of Tyr and Thr residues is required for the enzymatic activation of MAPKs, and the activation loop primarily contains the Thr-X-Tyr motif, where X can be any amino acid (aa) (2). The MAPK cascade is initiated by upstream signaling components, such as activated monomeric G protein and other protein kinases, which activate MAP3K. This, in turn, phosphorylates MAP2K, which subsequently phosphorylates MAPK to relay signals. The activated MAPK then phosphorylates downstream effectors or regulators, including transcription factors, kinases, phosphatases, and other effector proteins (3).

MAPK pathways play essential roles in the growth, differentiation, and pathogenicity of most plant and animal fungal pathogens. Fungi typically contain up to six MAPKs, but three major fungal MAPKs are widely conserved: (i) mating/filamentation MAPK, (ii) cell wall integrity (CWI) MAPK, and (iii) stress-activated MAPK (4). The mating/filamentation MAPK pathway regulates the mating process, which is stimulated by extracellular mating pheromones. Production of these pheromones is triggered by the G protein-coupled receptor and the mating/filamentation MAPK cascade. In the model budding yeast *Saccharomyces cerevisiae*, Fus3 and Kss1 are MAPKs of this class, activated by MAP2K Ste7 and MAP3K Ste11. Fus3 and Kss1 activate the HMG (high mobility group)-box transcription factor Ste12, which in turn induces the expression of mating-related genes. However, these two MAPKs have distinct roles: Fus3 is involved in cell cycle arrest and polarized growth toward the mating partner, while Kss1 regulates filamentous growth under nutrient-limited conditions (5, 6).

The CWI MAPK pathway primarily regulates cell wall homeostasis. In *S. cerevisiae*, Slt2 (also known as Mpk1) represents this MAPK class. Membrane proteins such as Mid2 and Wsc1 act as sensors when cell wall integrity is compromised. Protein kinase C (Pkc1) is activated when these membrane sensor proteins engage with the guanine nucleotide exchange factor Rom2, which in turn activates the Rho1 GTP-binding protein. Pkc1 activates the MAP3K Bck1, which phosphorylates two redundant MAP2Ks, Mkk1 and Mkk2. These MAP2Ks then activate the MAPK Slt2, which induces specific transcriptional responses via the Rim1 transcription factor. Additionally, Slt2 controls the mitotic cell cycle’s G_1_/S transition in a Rim1-independent manner (7, 8). Notably, *S. cerevisiae* also has a pseudo-kinase Slt2-like paralog Mlp1, which lacks a kinase domain but shares functions with Slt2 in cell cycle regulation, transcription activation, and interaction with MAPK phosphatase Msg5 (9, 10).

The stress-activated MAPK pathway is exemplified by the high-osmolarity glycerol (HOG) pathway, which is activated in response to various environmental stresses, such as hyperosmotic conditions, high temperatures, and oxidative stress. In *S. cerevisiae*, osmotic sensors Sho1 and Sln1 detect stress signals and activate MAP3Ks Ssk2/Ssk22 and Ste11, which in turn activate MAP2K Pbs2 and MAPK Hog1. Phosphorylated Hog1 translocates to the nucleus, activating three transcription factors: Sko1, Hot1, and Smp1. Activated Hog1 increases intracellular glycerol contents, regulates the cell cycle, and orchestrates other cellular responses (11, 12).

The basidiomycete human fungal pathogen *Cryptococcus neoformans* causes fatal meningoencephalitis, primarily in immunocompromised individuals, leading to 194,000 infection cases and 147,000 deaths worldwide annually (13). This opportunistic pathogen has five MAPK-like proteins, as identified in the *C. neoformans* H99 genome database. Among them, three primary MAPKs—Cpk1 (CNAG_02511, a Fus3/Kss1 ortholog), Mpk1 (CNAG_04514, a Slt2 ortholog), and Hog1 (CNAG_01523)—have been thoroughly characterized in *C. neoformans* (14). The Cpk1 MAPK pathway is sequentially activated by MAP2K Ste7 and MAP3K Ste11. Deletion of Ste11, Ste7, or Cpk1 results in defects in mating pheromone production, cell fusion, and filamentous growth but does not affect virulence (15). The Mpk1 MAPK pathway, involved in CWI maintenance, is activated by MAP2K Mkk1/2 and MAP3K Bck1. Inhibition of this pathway makes cells highly susceptible to cell wall/membrane, genotoxic and osmotic stresses, high temperatures, and antifungal drugs, reducing *C. neoformans* pathogenicity (16). Finally, the Hog1 MAPK pathway is sequentially activated by MAP2K Pbs2 and MAP3K Ssk2 and is essential for adapting to various environmental stresses and regulating melanin and capsule production, sexual differentiation, and ergosterol biosynthesis (14, 17).

In addition to the three major MAPKs, *C. neoformans* possesses Cpk2 (CNAG_02531) and Mpk2 (CNAG_04282), paralogs of Cpk1 and Mpk1, respectively, with their roles in *C. neoformans* remaining unclear. Our previous genome-wide functional surveys of cryptococcal kinases demonstrated that Cpk2 plays minor roles in osmotic and DNA damage stress responses and melanin production but is dispensable for the mating process, unlike Cpk1 (16). Deletion of *CPK2* does not result in virulence or infectivity defects in *C. neoformans* (16, 18). Similarly, Mpk2, unlike Mpk1, plays minor roles in cell membrane stress response, resistance to fludioxonil and fluconazole, and melanin and urease production (16). Mpk2 is essential to virulence in mice but not in insect models, distinguishing it from its paralog Mpk1 (16, 18).

This study aimed to elucidate the functional connections between Cpk2 and Mpk2 with the Cpk1- and Mpk1-dependent MAPK signaling pathways in *C. neoformans*. Consistent with their phylogenetic relationship, we provide experimental evidence demonstrating that Cpk2 and Mpk2 play redundant roles with Cpk1 and Mpk1, respectively. Most surprisingly, we found that deletion of both Mpk1 and Mpk2 completely restored normal mating in *cpk1*Δ mutants, indicating that Mpk1 and Mpk2 have overlapping roles in negatively regulating the Cpk1-dependent mating process in *C. neoformans*.

## RESULTS

### Phylogenetic and predicted structure analysis of cryptococcal MAPKs Cpk2 and Mpk2

**Fig 1:**
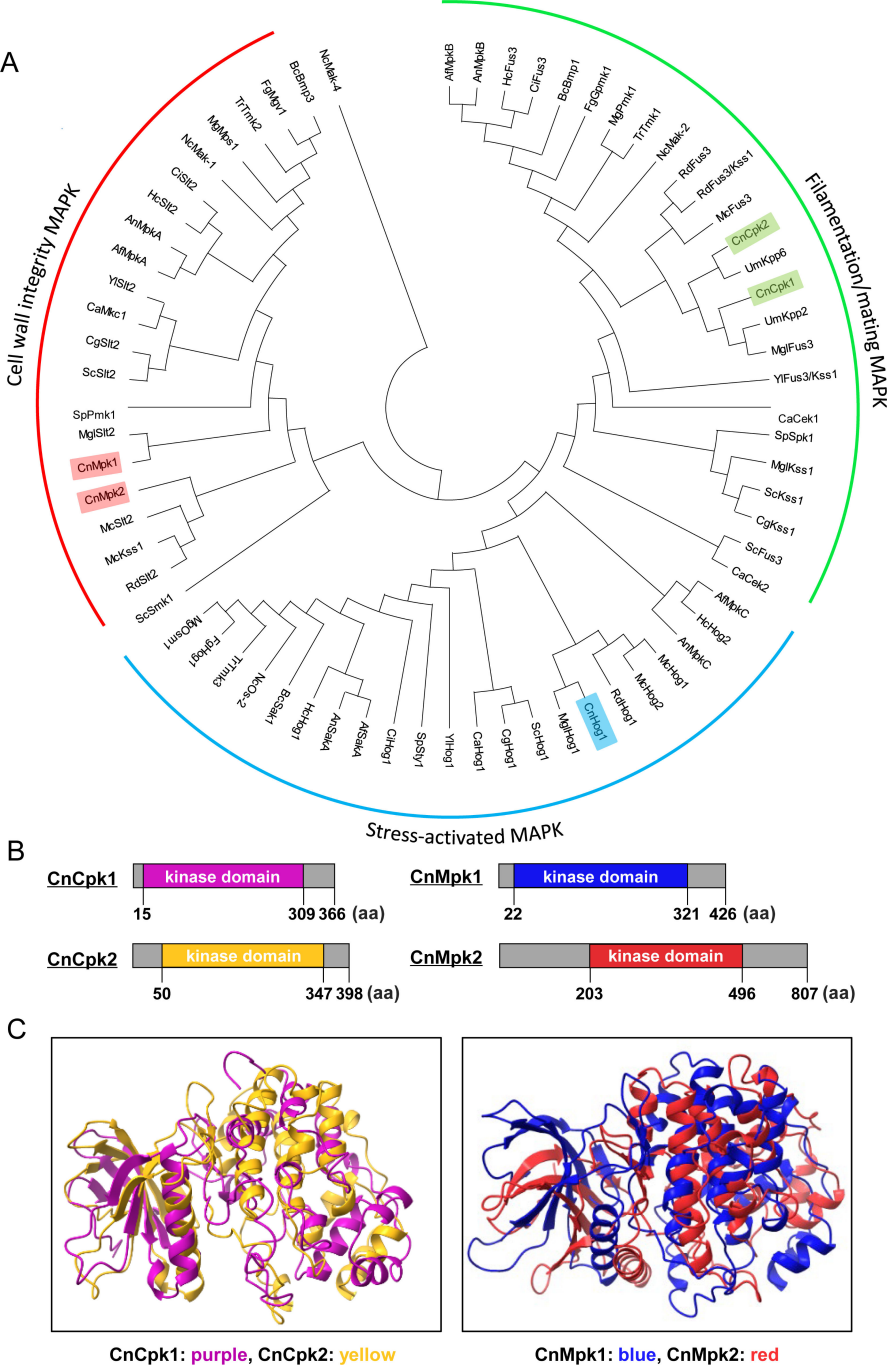
Phylogenetic and structural analyses of Cpk1/Cpk2 and Mpk1/Mpk2 MAPK orthologs in fungi. (**A**) A phylogenetic tree illustrating MAPKs from 19 distinct fungal species is presented. The species abbreviations are as follows: Cn for *C. neoformans*; Sc for *S. cerevisiae*; Sp for *Schizosaccharomyces pombe*; Ca for *Candida albicans*; Cg for *Candida glabrata*; Fg for *Fusarium graminearum*; Mg for *Magnaporthe grisea*; Nc for *Neurospora crassa*; Af for *Aspergillus fumigatus*; An for *Aspergillus niger*; Bc for *Botrytis cinerea*; Um for *Ustilago maydis*; Yl for *Yarrowia lipolytica*; Rd for *Rhizopus delemar*; Hc for *Histoplasma capsulatum*; Mgl for *Malassezia globosa*; Mc for *Mucor circinelloides*; Tr for *Trichoderma reesei*; and Ci for *Coccidioides immitis*. (**B and C**) Illustrations depict the protein structures of the cryptococcal MAPKs: Cpk1, Cpk2, Mpk1, and Mpk2. Kinase domains for Cpk1 (in purple), Cpk2 (in yellow), Mpk1 (in blue), and Mpk2 (in red) were predicted by the InterPro domain analysis (https://www.ebi.ac.uk/interpro) and shown alongside their respective paralogs. Their kinase domain structures were predicted using AlphaFold2’s MMseqs2-based protein structure prediction method. Structural comparisons between Cpk1/Cpk2 and Mpk1/Mpk2 were visualized using the Mol*3D Viewer at the RCSB Protein Data Bank.

In *C. neoformans* H99, five MAPKs (Cpk1, Cpk2, Mpk1, Mpk2, and Hog1) were identified with BLAST search. Based on phylogenetic analysis, all five cryptococcal MAPKs appeared to be canonical types of fungal MAPKs: Cpk1 and Cpk2 in the mating/filamentation-related MAPK clade, Mpk1 and Mpk2 in the CWI-related MAPK clade, and Hog1 for the stress response-related MAPK clade (Fig. 1A). The InterPro domain analysis indicates that Cpk1, comprising 366 aa, and Cpk2, consisting of 398 aa, are proteins of comparable size. Additionally, both proteins exhibit a protein kinase domain of similar size and location (Fig. 1B). Furthermore, Cpk1 and Cpk2 show highly similar kinase domain structures predicted by AlphaFold2 (Fig. 1C) (19). In contrast, Mpk2, with a size of 807 aa, is significantly larger than Mpk1, which consists of 426 aa. Notably, Mpk2 possesses extended N-terminal and C-terminal regions that are substantially longer than those found in Mpk1 (Fig. 1B). Nevertheless, protein kinase domain structures of Mpk1 and Mpk2, predicted by AlphaFold2, appeared to be highly similar to each other (Fig. 1C). Therefore, Cpk2 and Mpk2 appear to be phylogenetic and structural paralogs of Cpk1 and Mpk1, respectively, in *C. neoformans*.

### The cryptic roles of Cpk2 as a complementary kinase of Cpk1 in the mating process

We previously constructed the single MAPK mutants *cpk1*Δ, *cpk2*Δ, *mpk1*Δ, *mpk2*Δ, and *hog1*Δ and analyzed *in vitro* and *in vivo* phenotypic traits (14, 16, 17, 20, 21). To further investigate the cryptic functions of minor MAPKs, Cpk2 and Mpk2, in this study, we generated a series of double MAPK mutants of *C. neoformans: cpk1*Δ *cpk2*Δ, *mpk1*Δ *mpk2*Δ, *mpk1*Δ *cpk2*Δ, and *cpk2*Δ *mpk2*Δ (Fig. S1A).

**Fig 2:**
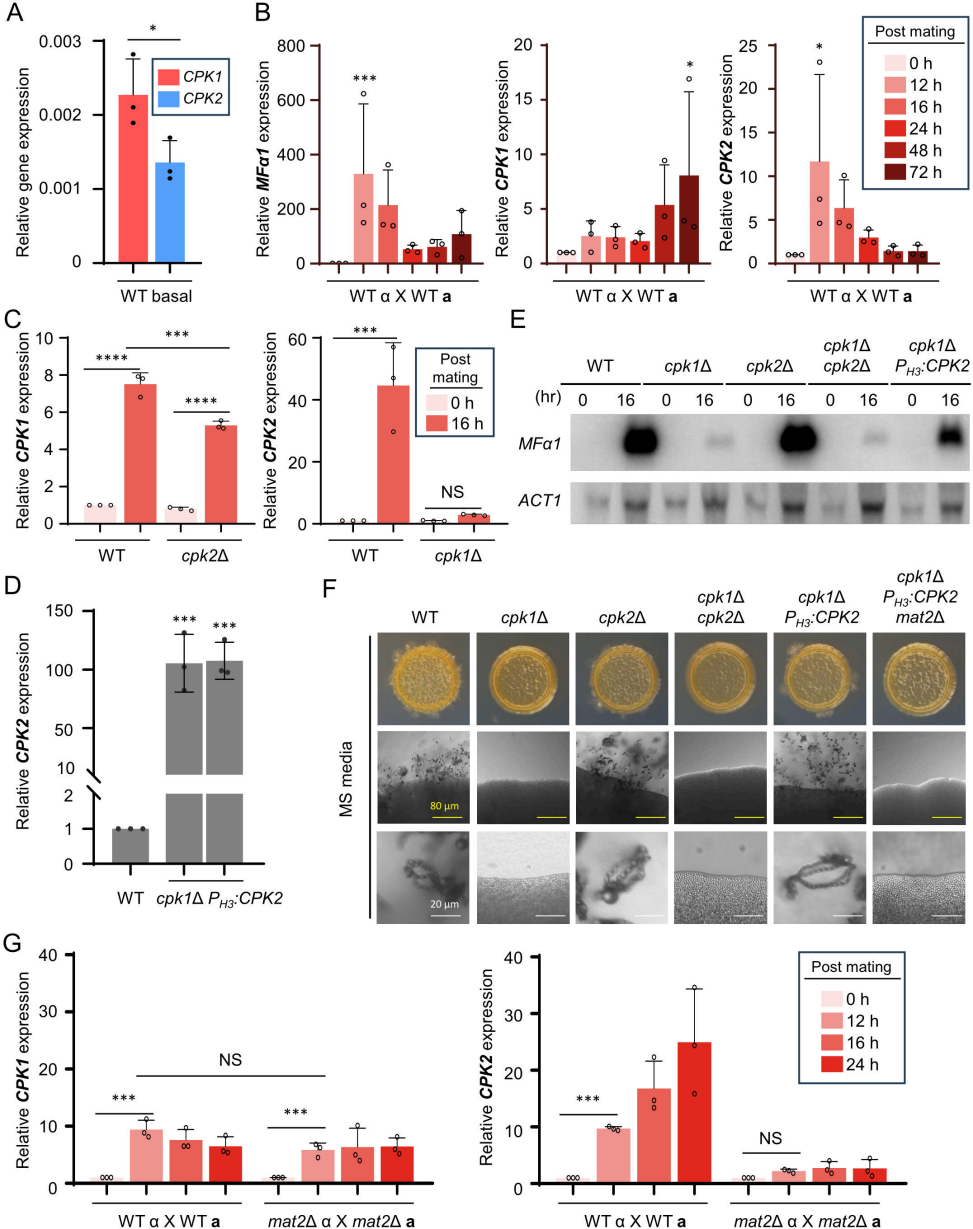
Cpk2 operates as a functional paralog to Cpk1 in *C. neoformans*. (**A**) The expression level of *CPK1* and *CPK2* was quantified relative to that of *ACT1* using a quantitative reverse transcription-PCR (qRT-PCR) assay in a pre-mating wild-type sample. (**B**) In *C. neoformans* serotype A, *MAT*α and *MAT***a** wild-type strains underwent bisexual mating (H99 × YL99**a**) in V8 medium at room temperature in the dark. After 0, 12, 16, 24, 48, and 72 h post-mating, expression levels of *MFα1*, *CPK1*, and *CPK2* were analyzed through qRT-PCR. (**C**) After unilateral mating of wild-type (H99 × YL99**a**) and *cpk1*Δ (*cpk1*Δ *MAT*α × YL99**a**) strains, cells were collected at the specified time points. RNA from these cells was used to determine *CPK2* expression via qRT-PCR. Similarly, following mating with wild-type and *cpk2*Δ (*cpk2*Δ *MAT*α × YL99**a**) strains, *CPK1* expression was assessed. (**D**) *CPK2* expression was quantified in the wild-type (H99) and *cpk1*Δ P*_H3_:CPK2* (YSB9414 and YSB9419) strains. Validation involved three independent qRT-PCR assays. (**E**) YL99**a**, along with H99 and *MAT*α mutant strains—*cpk1*Δ (YSB127), *cpk2*Δ (YSB373), *cpk1*Δ *cpk2*Δ (YSB8126), and *cpk1*Δ P*_H3_:CPK2* (YSB9414)—were cultivated. After 16 h post-mating, cells were harvested and subjected to RNA extraction. The expression of *MFα1* was measured via northern blot analysis. Post-analysis, the membrane was reprobed to measure actin gene (*ACT1*) expression. (**F**) YL99**a**, alongside H99 and knockout mutants *cpk1*Δ, *cpk2*Δ, *cpk1*Δ P*_H3_:CPK2*, and *cpk1*Δ P*_H3_:CPK2 mat2*Δ (YSB10284), were cultured. Equal proportions of *MAT*α and *MAT***a** wild-type cells were co-spotted on MS (Murashige and Skoog) media. After 11 days, morphological changes, including colony morphology, filamentation, and sporulation, were documented. (**G**) Wild-type (H99 and YL99**a**) and *mat2*Δ [*mat2*Δ *MAT*α (YSB10585) and *mat2*Δ *MAT***a** (YSB10577)] strains were cultivated and plated on V8 media for bilateral mating. After 0, 12, 16, and 24 h post-mating, cells were harvested, RNA was extracted, and expression levels of *CPK1* and *CPK2* were determined through qRT-PCR. The data represent three biological and three technical repetitions. Statistical analysis was conducted using one-way analysis of variance (ANOVA) with Tukey’s test: *, *P* < 0.05; **, *P* < 0.01; ***, *P* < 0.001; ****, *P* < 0.0001; NS = not significant.

It is well known that Cpk1 is the dominant kinase that governs the mating pheromone MAPK pathway (15). Regardless of the phylogenetic and structural relationship between Cpk1 and Cpk2, *cpk2*Δ mutants do not show any mating defects, unlike *cpk1*Δ mutants (16). As *CPK1* deletion alone abolishes pheromone production, cell-to-cell fusion, and filamentation, it was impossible to observe additional mating defects in *cpk1*Δ *cpk2*Δ mutants. Therefore, to find any potential roles of *CPK2* in mating, we examined whether *CPK2* expression is induced during mating. Under pre-mating condition, the basal expression level of *CPK2* was slightly but significantly lower than that of *CPK1* (Fig. 2A). When *MAT*α *C. neoformans* strain (H99) was co-cultured with its isogenic *MAT***a** *C. neoformans* strain (YL99**a**), expression of mating pheromone α gene (*MFα1*) was highly induced after 12–72 h post-mating. Similarly, *CPK1* expression was induced after 12–72 h post-mating. Notably, *CPK2* expression was also highly induced after 12–72 h post-mating (Fig. 2B), implying that Cpk2 may play a role in mating in *C. neoformans*.

To explore whether Cpk1 and Cpk2 transcriptionally regulate each other, we performed unilateral mating using *MAT*α *cpk1*Δ and *cpk2*Δ mutants. Under mating conditions, both the wild-type and *cpk2*Δ strains showed increased expression levels of *CPK1*; however, the level of this increase was significantly smaller in *cpk2*Δ (approximately fivefold induction) than in the wild type (approximately eightfold induction), suggesting that Cpk2 may play a role in inducing *CPK1* during mating. Conversely, we discovered that Cpk1 plays a dominant role in *CPK2* expression induction during mating, which was nearly abolished in *cpk1*Δ (Fig. 2C).

To address whether Cpk2 plays a redundant role with Cpk1, we constructed two independent *CPK2* overexpression mutants in the *cpk1*Δ background by replacing the native promoter of *CPK2* with the histone 3 (*H3*) promoter (*cpk1*Δ P*_H3_:CPK2*; Fig. S1B). We verified that *CPK2* was successfully overexpressed over 100-fold in *cpk1*Δ P*_H3_:CPK2* than in the wild type (Fig. 2D). As reported before, a lack of pheromone-responsive MAPK Cpk1 almost abolished pheromone expression (Fig. 2E), filamentation, and sporulation (Fig. 2F). It is noteworthy to highlight that the overexpression of *CPK2* in the *cpk1*Δ mutant substantially reinstated *MFα1* induction (Fig. 2E), mating filamentation, and sporulation in the *cpk1*Δ strain (Fig. 2F), thereby suggesting the potential for Cpk2 to act as a complementary MAPK to Cpk1 in facilitating mating processes when overexpressed.

We further investigated if *CPK2* overexpression enhances *cpk1*Δ mating in a Cpk1-dependent or -independent manner by deleting the *MAT2* gene in *cpk1*Δ P*_H3_:CPK2*. Mat2 is a central HMG transcription factor operating downstream of the Cpk1 MAPK, controlling the expression of mating-related genes (22). Additional deletion of *MAT2* abolished the recovered mating phenotypes in the *cpk1*Δ P*_H3_:CPK2* strain (Fig. 2F), indicating that Cpk2 operates in a Cpk1- and Mat2-dependent manner. To determine if it provides feedback regulation to Cpk1 or Cpk2, we conducted bilateral mating experiments using *MAT*α and *MAT***a** *mat2*Δ mutants. *CPK1* induction levels were similar in wild-type and *mat2*Δ strains, indicating that Mat2 is not involved in mating-dependent *CPK1* induction. However, *CPK2* induction did not occur in *mat2*Δ bilateral mating, indicating that Mat2 promotes the induction of *CPK2* expression through a positive feedback reaction (Fig. 2G). Collectively, these findings imply that Cpk2 may have the potential to functionally substitute for Cpk1 in the sexual developmental processes of *C. neoformans*.

### The cryptic roles of Mpk2 as a complementary kinase of Mpk1 in the CWI pathway

**Fig 3:**
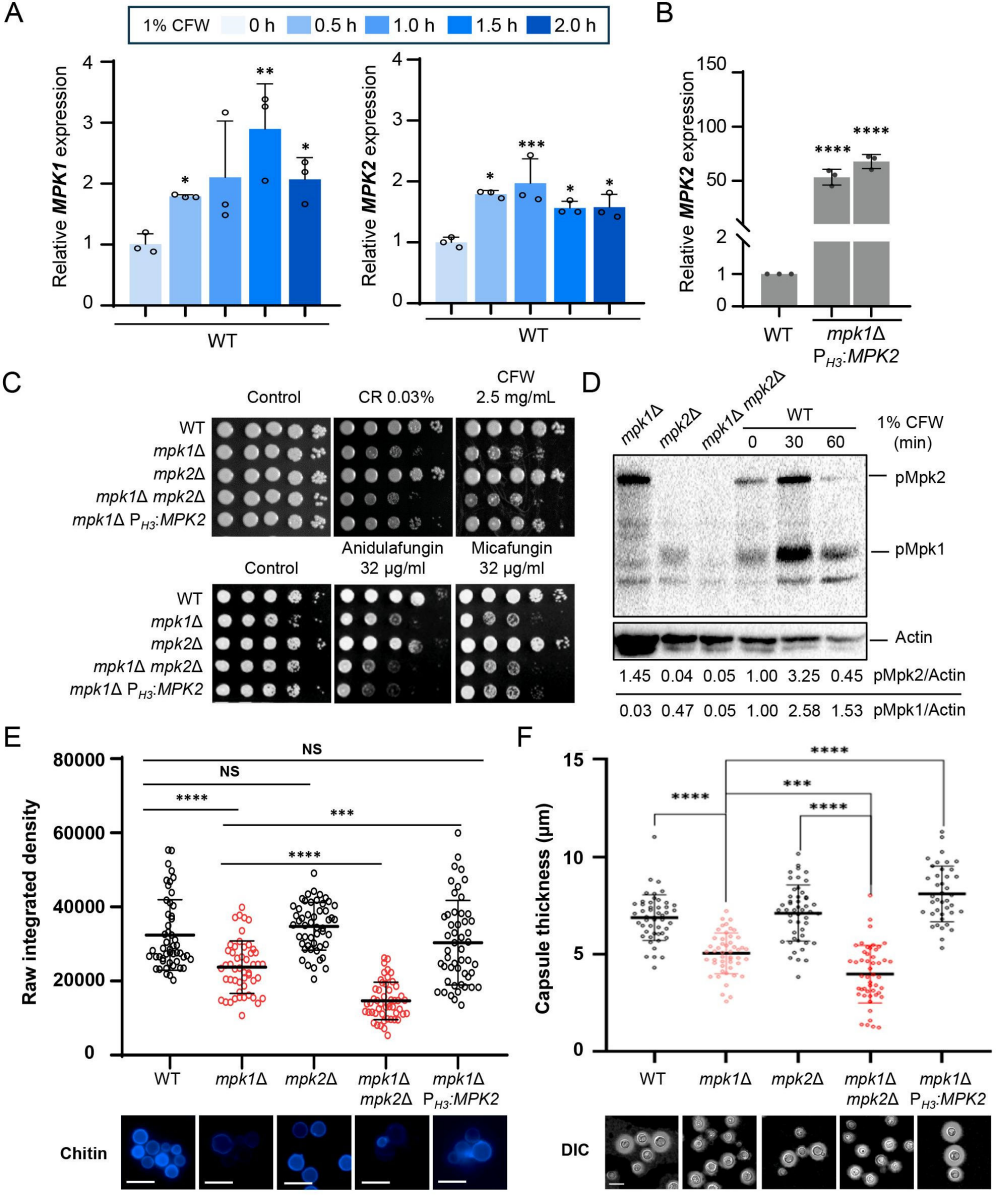
Mpk2 functions as an Mpk1 paralog for maintaining the CWI. (**A**) The wild-type strain (H99) was grown to an OD_600_ of 0.8 before transitioning to a fresh yeast extract-peptone-dextrose (YPD) medium containing 1% CFW. Cells were harvested at intervals of 0, 30, 60, 90, and 120 min and subjected to RNA extraction and cDNA synthesis. Expression levels of *MPK1* and *MPK2* were determined via quantitative reverse transcription-PCR (qRT-PCR) in three biological replicates. (**B**) *MPK2* expression levels were quantified by qRT-PCR with three biological replicates in the wild-type (H99) and *mpk1*Δ P*_H3_:MPK2* (YSB9585 and YSB9586) strains. (**C**) Following overnight growth, cells from wild-type (H99), *mpk1*Δ (KK3), *mpk2*Δ (YSB3236), *mpk1*Δ *mpk2*Δ (YSB8245), and *mpk1*Δ P*_H3_:MPK2* (YSB9575) strains were serially diluted to 10^−4^ and spotted on solid media containing various stressors: 0.03% Congo Red (CR), 2.5 mg/mL CFW, 32 µg/mL anidulafungin, or 32 µg/mL micafungin. After 2 days, the plates were imaged. (**D**) Wild-type, *mpk1*Δ, *mpk2*Δ, and *mpk1*Δ *mpk2*Δ strains were cultured overnight, synchronized to OD_600_ 0.2, and further grown to 0.8 with fresh YPD. The strains were then treated with 1% CFW for 0, 30, and 60 min. After specified times, 40 mL of cells was collected and frozen with liquid nitrogen. Whole proteins were extracted and immunoblotted with phospho-p44/42 antibody. Phosphorylated Mpk1 and Mpk2 levels were measured using the chemiluminescence method, after which the membranes were stripped and reprobed with anti-actin antibody to quantify total proteins. Protein quantification was conducted using the ImageLab program. (**E**) Wild-type, *mpk1*Δ, *mpk2*Δ, *mpk1*Δ *mpk2*Δ, and *mpk1*Δ P*_H3_:MPK2* strains were cultured overnight and adjusted to an OD_600_ of 0.2 and then grown for 24 h in YPD at 30°C and fixed with formaldehyde solution. Cells were stained with CFW in the dark, washed, and imaged using differential interference contrast (DIC) and DAPI (4′,6-diamidino-2-phenylindole) filters. Chitin content in the membrane of randomly chosen 50 cells was assessed using ImageJ. (**F**) The aforementioned strains in panels D and E were grown in capsule-inducing Littman medium for 2 days. Cells were then stained with India ink, and the capsular thickness of 50 randomly selected cells was measured using NIS-elements software. The indicated white scale bar is 10 µm. Statistical differences were determined using one-way ANOVA and Tukey’s test: *, *P* < 0.05; **, *P* < 0.01; ***, *P* < 0.001; ****, *P* < 0.0001; NS = not significant.

The CWI pathway in *C. neoformans* is reported to be regulated by Mpk1 (23). The deletion of *MPK1* leads to increased susceptibility to cell wall stress and cell wall destabilizing antifungal agents (23). Considering that Mpk2 appears to be a structural paralog of Mpk1, we addressed whether Mpk2 plays functionally redundant roles with Mpk1. First, we examined the expression of *MPK1* and *MPK2* upon cell wall stress. *MPK1* and *MPK2* expressions were elevated by the cell wall disrupting agent calcofluor white (CFW). These increases were about threefold and twofold, respectively, suggesting that Mpk2 may regulate the CWI pathway like Mpk1 (Fig. 3A).

Next, we generated mutants overexpressing *MPK2* in the background where the *MPK1* gene was deleted. To achieve this, we replaced the native promoter of the *MPK2* gene with the *H3* promoter, resulting in the creation of two independent mutants (*mpk1*Δ P*_H3_:MPK2*; Fig. S1B). Subsequently, we verified that these mutants exhibited an *MPK2* overexpression of more than 50-fold (Fig. 3B). Then, we compared growth phenotypes of *mpk1*Δ, *mpk2*Δ, *mpk1*Δ *mpk2*Δ, and *mpk1*Δ P*_H3_:MPK2* strains under cell wall integrity-perturbing stresses, such as Congo red (CR), CFW, and cell wall destabilizing antifungal agents, such as micafungin and anidulafungin (Fig. 3C). We found that the deletion of *MPK2* did not lead to any alteration in susceptibility to these stresses and antifungal agents, and the *mpk1*Δ *mpk2*Δ mutant was phenotypically similar to the *mpk1*Δ mutant. Notably, however, we found that *MPK2* overexpression partially restored resistance to cell wall stresses (CR and CFW; Fig. 3C; Fig. S2A), indicating that Mpk2 may play redundant roles with Mpk1 in the CWI pathway. To acquire further evidence, we monitored the phosphorylation patterns of Mpk1 and Mpk2 in response to CFW. We discovered that a phospho-p44/42 (Erk1/2) antibody can detect the phosphorylation status of both Mpk1 and Mpk2 (Fig. 3D). Consistent with previous studies, we confirmed an increase in the phosphorylation of Mpk1 in response to CFW treatment (23). Notably, we also found that Mpk2 became more phosphorylated in response to CFW (Fig. 3D; Fig. S2B), suggesting that Mpk1 and Mpk2 play redundant roles in responding to cell wall stress in *C. neoformans*.

The redundant roles of Mpk1 and Mpk2 in cell wall stress resistance prompted us to evaluate their roles in the integrity of cell wall components, such as chitin and polysaccharide capsules. The loss of Mpk1 led to a significant reduction in chitin composition; however, *MPK2* overexpression restored it to levels comparable to the wild type. Moreover, chitin content declined in *mpk1*Δ *mpk2*Δ even more than in *mpk1*Δ, indicating that Mpk1 and Mpk2 are both involved in regulating chitin content (Fig. 3E). The polysaccharide capsule, which is the outermost cell wall layer and crucial virulence factor in *C. neoformans*, has previously been reported to be reduced in *mpk1*Δ (16). As expected, we observed that *MPK2* overexpression restored normal capsules in *mpk1*Δ. Furthermore, akin to the chitin component results, the *mpk1*Δ *mpk2*Δ mutant displayed an even greater reduction in capsule size compared to *mpk1*Δ (Fig. 3H). All these data supported that Mpk2 partially complements the functions of Mpk1 by regulating chitin content, capsule production, and cell wall stress response, thus confirming Mpk’s status as a functional paralog of Mpk1 for cell wall integrity in *C. neoformans*.

### The Mpk2 MAPK is controlled by the Mkk2 MAP2K and Ssk2/Ste11 MAP3Ks

**Fig 4:**
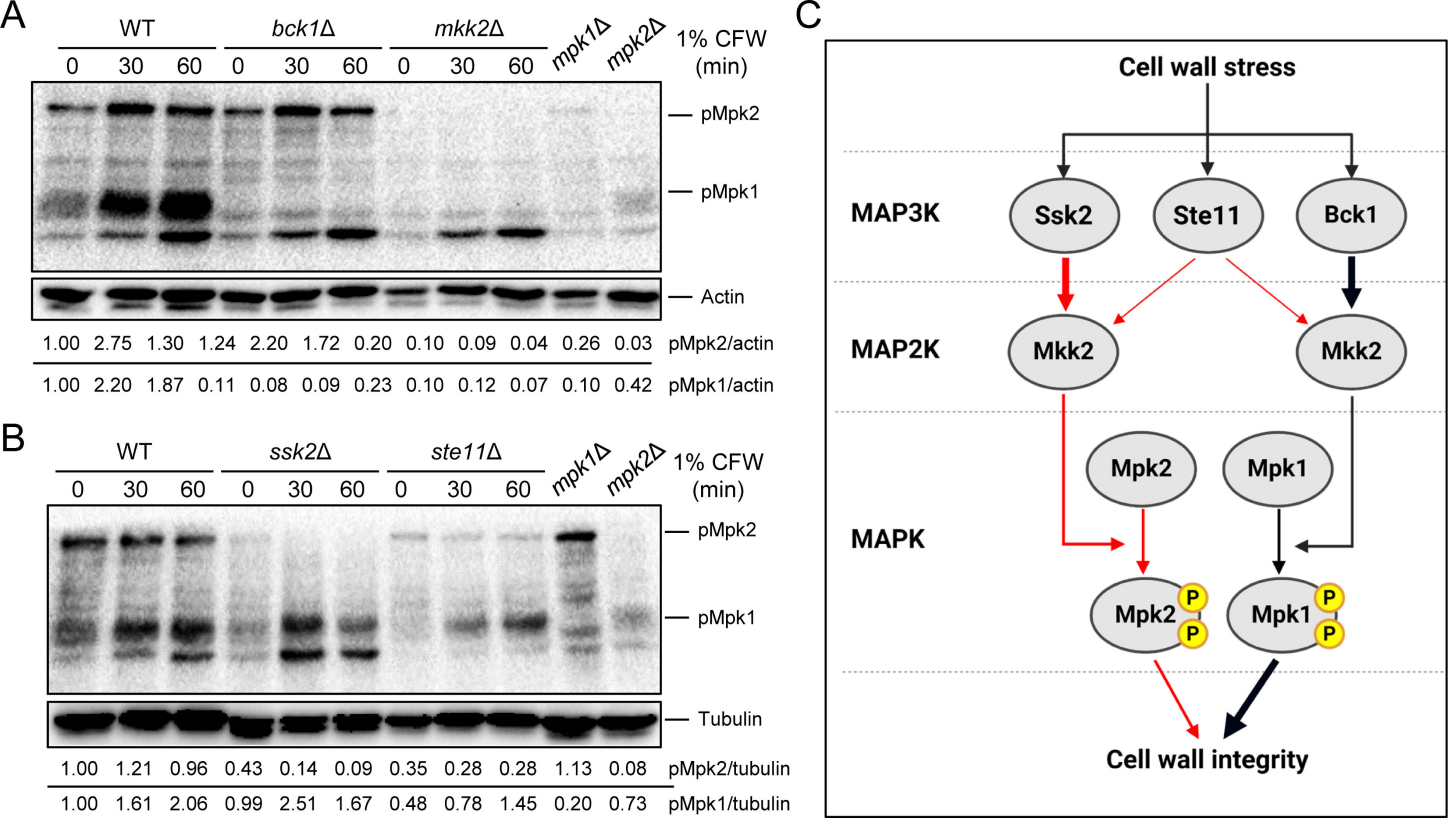
Mpk2 is regulated by MAP2K Mkk2 and MAP3Ks Ssk2 and Ste11. (**A and B**) Wild-type, *mpk1*Δ, *mpk2*Δ, *bck1*Δ (YSB273), *mkk2*Δ (YSB330), *ssk2*Δ (YSB264), and *ste11*Δ (YSB313) strains were grown overnight, resuspended with fresh yeast extract-peptone-dextrose (YPD) medium at an OD_600_ of 0.2, and further incubated to an OD of 0.8. Subsequently, these strains underwent a treatment with 1% CFW for intervals of 0, 30, and 60 min. Following each designated time point, 40 mL of the cell cultures was spun down and flash-frozen in liquid nitrogen. Total proteins were then extracted for immunoblot analysis using an anti phospho-p44/42 antibody. The levels of phosphorylated Mpk1 and Mpk2 were detected by chemiluminescence. After detection, the blots were stripped and re-probed for actin or tubulin to assess the total protein levels. (**C**) A schematic of the proposed Mpk1/Mpk2 regulatory mechanisms for maintaining CWI in *C. neoformans*.

In our subsequent analysis, we investigated whether the MAP2K Mkk2 and the MAP3K Bck1, upstream regulators known to activate Mpk1, also govern the phosphorylation and activation of Mpk2. Western blot analysis showed a complete loss of Mpk1 and Mpk2 phosphorylation in the *mkk2*Δ mutant (Fig. 4A), confirming Mkk2’s role in regulating both MAPKs. Intriguingly, while Mpk1 phosphorylation was absent in the *bck1*Δ mutant, mirroring the *mkk2*Δ result, Mpk2 phosphorylation was unaffected in *bck1*Δ and resembled the wild-type response to CFW (Fig. 4A; Fig. S3A). This finding led us to conclude that Mpk2 is not regulated by Bck1 within the CWI MAPK pathway.

We then considered alternative MAP3Ks, such as Ssk2 of the HOG pathway and Ste11 of the pheromone-responsive pathway, as potential regulators of Mpk2. Surprisingly, Mpk2 phosphorylation was markedly reduced in both *ssk2*Δ and *ste11*Δ mutants, whereas Mpk1 phosphorylation closely resembled that of the wild-type strain (Fig. 4B; Fig. S3B). These results collectively suggest that Ssk2 and Ste11 may act as upstream MAP3Ks that target the Mkk2 MAP2K, thereby modulating Mpk2 phosphorylation (Fig. 4C).

### Mpk1 and Mpk2 have distinct roles in maintaining cell membrane integrity

**Fig 5:**
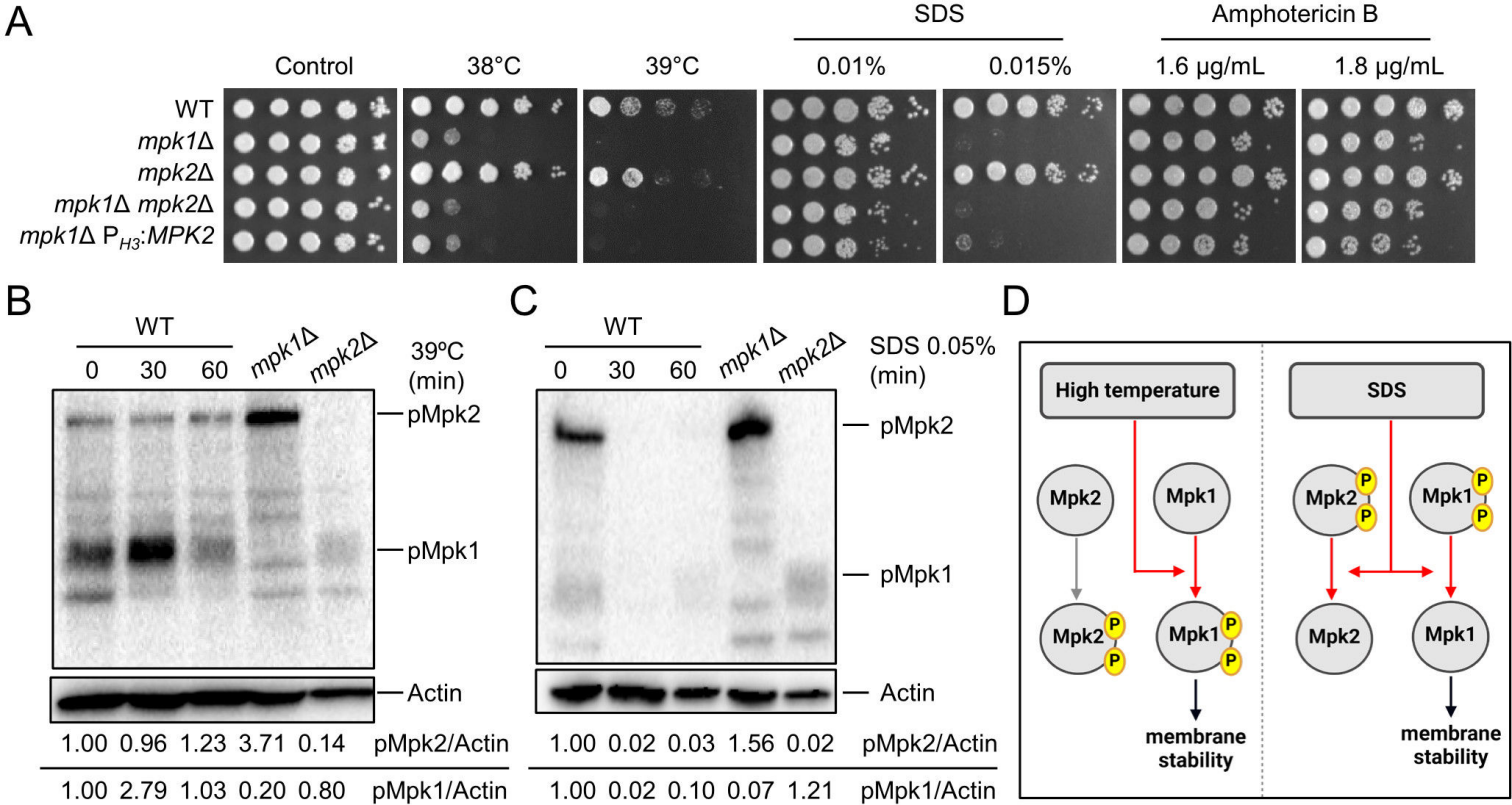
Mpk1 and Mpk2 play distinct roles in maintaining cell membrane integrity. (**A**) Wild-type, *mpk1*Δ, *mpk2*Δ, *mpk1*Δ *mpk2*Δ, and *mpk1*Δ P*_H3_:MPK2* strains were cultured overnight and then subjected to spot assays under various membrane stresses including high temperature, SDS, and amphotericin B. The photos were taken on day 3. (**B and C**) Wild-type, *mpk1*Δ, and *mpk2*Δ strains were cultured overnight and subcultured to an OD_600_ of 0.8 before being treated with high temperature at 39°C or 0.05% SDS. After the treatment, samples were harvested at 0, 30, and 60 min and frozen in liquid nitrogen before protein extraction. The extracted proteins were quantified, loaded in equal amounts, and subjected to western blot to assess the levels of phosphorylated Mpk1 and Mpk2. Additionally, after stripping the membrane, reprobing was conducted with an anti-actin antibody to quantify the total protein. (**D**) A schematic of the proposed Mpk1/Mpk2 regulatory mechanisms for maintaining cell membrane integrity in *C. neoformans*

Our findings that the phosphorylation of Mpk1 and Mpk2 is activated by distinct MAP3Ks suggest that Mpk1 and Mpk2 may have different biological roles in *C. neoformans*. It is established that the Mpk1 MAPK pathway also contributes to cell membrane integrity (16). Therefore, we investigated whether Mpk2 could similarly complement Mpk’s function in cell membrane stress response. We conducted a comparative phenotypic analysis on *mpk1*Δ, *mpk2*Δ, *mpk1*Δ *mpk2*Δ, and *mpk1*Δ P*_H3_:MPK2* strains under diverse cell membrane integrity-perturbing stresses. Notably, neither the double deletion of *MPK1* and *MPK2* nor overexpression of *MPK2* affected the *mpk1*Δ phenotype in response to high temperature, SDS, or amphotericin B (Fig. 5A). Upon shift to 39°C from 30°C, only Mpk1 was phosphorylated, not Mpk2, underscoring Mpk’s sole role in responding to high-temperature stress (Fig. 5B). Interestingly, unlike high-temperature stress, SDS treatment did not induce phosphorylation of both Mpk1 and Mpk2 and indeed rather reduced their basal phosphorylation levels, demonstrating that the role of Mpk1 did not appear to depend on its phosphorylation (Fig. 5C). Mpk1 and Mpk2 are phosphorylated differently by various cell membrane stresses and play distinct roles in maintaining cell membrane integrity (Fig. 5D).

### Mpk1 and Mpk2 cooperate for the negative regulation of the Cpk1 MAPK pathway

**Fig 6:**
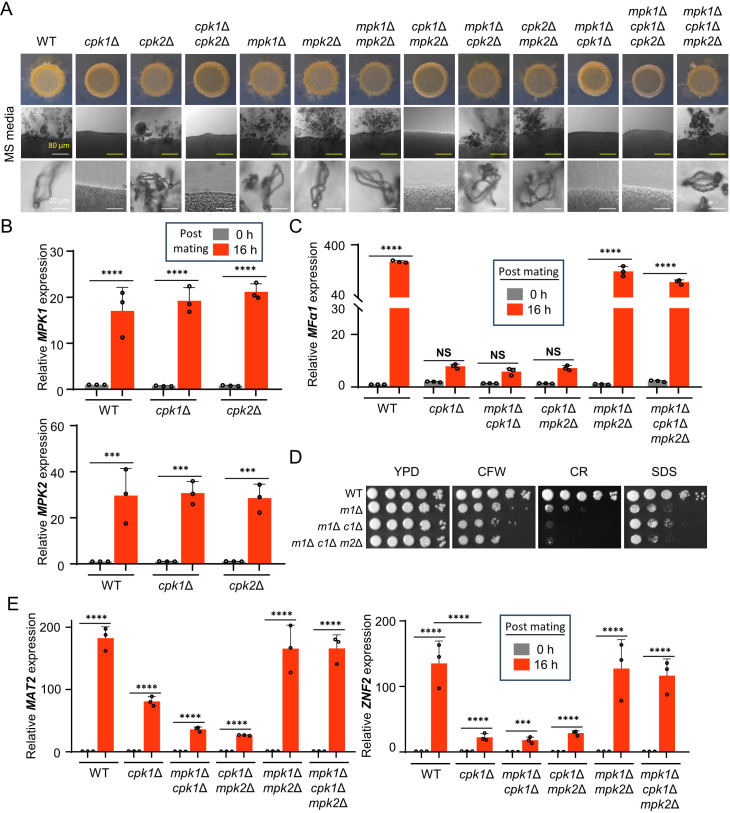
Mpk1 and Mpk2 cooperate for the negative regulation of the Cpk1 MAPK pathway. (**A**) The *MAT***a** wild-type YL99**a** strain was cultured with the following *MAT*α strains: wild type (H99), *cpk1*Δ, *cpk2*Δ, *mpk1*Δ, *mpk2*Δ, *cpk1*Δ *cpk2*Δ, *mpk1*Δ *mpk2*Δ, *cpk1*Δ *mpk2*Δ (YSB9206), *mpk1*Δ *cpk2*Δ (YSB8636), *cpk2*Δ *mpk2*Δ (YSB9584), *mpk1*Δ *cpk1*Δ (YSB6090), *mpk1*Δ *cpk1*Δ *cpk2*Δ (YSB10664), and *mpk1*Δ *cpk1*Δ *mpk2*Δ (YSB10643). Equal quantities of cells (10^7^) were mixed and spotted onto the mating-inducing MS medium. After 11 days of incubation at room temperature in the dark, colony morphology, filamentation, and spore formation were observed under a brightfield microscope. (**B**) *MAT*α wild-type, *cpk1*Δ, and *cpk2*Δ strains were subjected to unilateral mating with *MAT***a** wild-type on V8 medium. Following 0 and 16 h of incubation, RNA was extracted, cDNA was synthesized, and expression levels of *MPK1* and *MPK2* were quantified. (**D**) The wild-type and MAPK mutant strains were cultured and spotted under cell wall and membrane stress conditions using 0.05% CR, 0.5 mg/mL CFW, and 0.01% SDS. After 2 days, the plates were photographed. *m1*Δ refers *mpk1*Δ, *m1*Δ *c1*Δ refers *mpk1*Δ *cpk1*Δ, and *m1*Δ *c1*Δ *m2*Δ refers *mpk1*Δ *cpk1*Δ *mpk2*Δ. (**C** and **E**) *MAT*α wild-type, *cpk1*Δ, *mpk1*Δ *cpk1*Δ, and *mpk1*Δ *cpk1*Δ *mpk2*Δ strains underwent unilateral mating with the *MAT***a** wild-type YL99**a** strain. Post 0 and 16 h of incubation, RNA was extracted, and cDNA was synthesized. Expression of *MFα1*, *MAT2*, and *ZNF2* was quantified by quantitative reverse transcription-PCR (qRT-PCR) with three biological replicates. Statistical significance of the data was determined using one-way ANOVA with Tukey’s multiple-comparison test: *, *P* < 0.05; **, *P* < 0.01; ***, *P* < 0.001; ****, *P* < 0.0001; NS = not significant.

In *C. neoformans*, the CWI MAPK pathway aids in triggering cell wall gene activation and intracellular ATP delivery, thus permitting Gis1-reliant cell wall reactions and glucosamine-induced filamentation when responding to environmental cues (24). Moreover, previous phosphoproteomics data demonstrated that Mpk1 modulates Gis1 expression through the phosphorylation of Skn7 and Crz1, culminating in Znf2 activation, which drives the yeast-to-hypha transition (24). Furthermore, it is known that bilateral mating of *mpk2*Δ in *C. neoformans* showed a mating defect in V8 media (18). Given these previous data, we hypothesized that Mpk1 and Mpk2 could regulate sexual development with the Cpk1 MAPK pathway. To test this, we generated a series of *MAT*α double and triple knockout strains for *CPK1*, *CPK2*, *MPK1*, and *MPK2* and assessed their ability to undergo filamentation upon unilateral mating with YL99**a** strain. We observed that *cpk1*Δ *cpk2*Δ, *cpk1*Δ *mpk2*Δ, *mpk1*Δ *cpk1*Δ, and *mpk1*Δ *cpk1*Δ *cpk2*Δ were unable to form any filament, same as *cpk1*Δ (Fig. 6A). In contrast, *mpk1*Δ *mpk2*Δ, *mpk1*Δ *cpk2*Δ, and *cpk2*Δ *mpk2*Δ exhibited wild-type level of filamentation in Murashige and Skoog (MS) or V8 media, same as the individual single knockout mutants. Surprisingly, however, we found that *mpk1*Δ *cpk1*Δ *mpk2*Δ triple mutants completely restored the filamentation in wild-type levels (Fig. 6A; Fig. S4A). We generated three independent *mpk1*Δ *cpk1*Δ *mpk2*Δ strains, all of which restored normal filamentation like the wild type, indicating that it was not a mutational artifact. The fact that both *mpk1*Δ *cpk1*Δ and *mpk2*Δ *cpk1*Δ exhibited mating defects comparable to that of *cpk1*Δ strongly indicates that Mpk1 or Mpk2 alone is insufficient to negatively regulate downstream factors of the Cpk1 MAPK pathway during mating.

If Mpk1 and Mpk2 work as negative mating regulators, we hypothesized that *MPK1* and *MPK2* may undergo transcriptional regulation during mating. In support of this hypothesis, expression levels of *MPK1* and *MPK2* increased by about 20- and 30-fold, respectively, 16 h-post-mating of wild-type strains (Fig. 6B). Notably, the mating-dependent induction of *MPK1* and *MPK2* expression was similarly observed in *cpk1*Δ and *cpk2*Δ mutants, suggesting that such event appeared to be controlled in Cpk1- and Cpk2-independent manners.

To investigate the unexpected restoration of sexual development in *mpk1*Δ *cpk1*Δ *mpk2*Δ, we first examined the expression of the mating pheromone *MFα1* gene. The expression of *MFα1* was significantly reduced only in *cpk1*Δ, *mpk1*Δ *cpk1*Δ, and *cpk1*Δ *mpk2*Δ, which have severe mating defects. In contrast, *mpk1*Δ *cpk1*Δ *mpk2*Δ triple mutants exhibited restored *MFα1* expression to wild-type levels (Fig. 6C), suggesting that the wild-type-like filamentation observed in *mpk1*Δ *cpk1*Δ *mpk2*Δ was due to the induction of mating pheromone at levels comparable to those in wild-type strains.

To ascertain whether the recovery of the mating defect in *mpk1*Δ *cpk1*Δ *mpk2*Δ was not due to perturbed cell wall integrity, we exposed the *mpk1*Δ *cpk1*Δ *mpk2*Δ, along with the parental *mpk1*Δ *cpk1*Δ, to the cell wall and membrane stress agents. We confirmed that the triple knockout was as susceptible to CFW, CR, or SDS as the parental double mutant strain, thereby verifying that the recovery of the mating defect was not due to alteration of cell wall integrity (Fig. 6D). Additionally, the *mpk1*Δ *cpk1*Δ *cpk2*Δ and its parental *mpk1*Δ *cpk1*Δ strain showed similar susceptibility to CFW, CR, and SDS and mating defects, suggesting that *CPK2* deletion does not further influence cell wall integrity or the mating efficiency in *mpk1*Δ *cpk1*Δ (Fig. 6A; Fig. S4).

We hypothesized that potential Mpk1/Mpk2-regulated targets for mating are Mat2 and Znf2 because these are well-known downstream transcription factors of the Cpk1 MAPK pathway (22). Therefore, we checked the induction levels of *MAT2* and *ZNF2* expression post-mating. We confirmed that the transcription levels of both *MAT2* and *ZNF2* were strongly induced 16 h post-mating in wild type, while those were reduced by *CPK1* deletion, which was observed in *cpk1*Δ, *mpk1*Δ *cpk1*Δ, and *cpk1*Δ *mpk2*Δ with all showing mating defects. However, the wild-type induction levels of *MAT2* and *ZNF2* were recovered in the *mpk1*Δ *cpk1*Δ *mpk2*Δ (Fig. 6E). These data indicate that *MAT2* and *ZNF2* expression could be induced post-mating in a Cpk1-independent manner when both Mpk1 and Mpk2 were absent.

In addition to Mat2 and Znf2, Ste12α, which is the ortholog of the Ste12 transcription factor in the filamentation MAPK pathway in *S. cerevisiae*, could be another Mpk1/Mpk2-regulated target for mating because its deletion was shown to cause modest mating defects in *C. neoformans* (25–27). In support of the previous finding, we found that the expression of *STE12*α was induced post-mating in the wild type, while its induction was reduced in *cpk1*Δ. In contrast, the wild-type induction level of *STE12*α expression was restored in *mpk1*Δ *cpk1*Δ *mpk2*Δ, indicating that Mpk1 and Mpk2 could influence Ste12. However, considering that *mpk1*Δ *cpk1*Δ and *mpk2*Δ *cpk1*Δ, which showed mating defects, also had restored *STE12*α induction, the recovery of mating in *mpk1*Δ *cpk1*Δ *mpk2*Δ did not seem to be due to Ste12α (Fig. S4C). Collectively, our data demonstrated that Mpk1 and Mpk2 cooperate to negatively regulate the induction of two major Cpk1-dependent transcription factors, Mat2 and Znf2, during mating in *C. neoformans*.

## DISCUSSION

**Fig 7:**
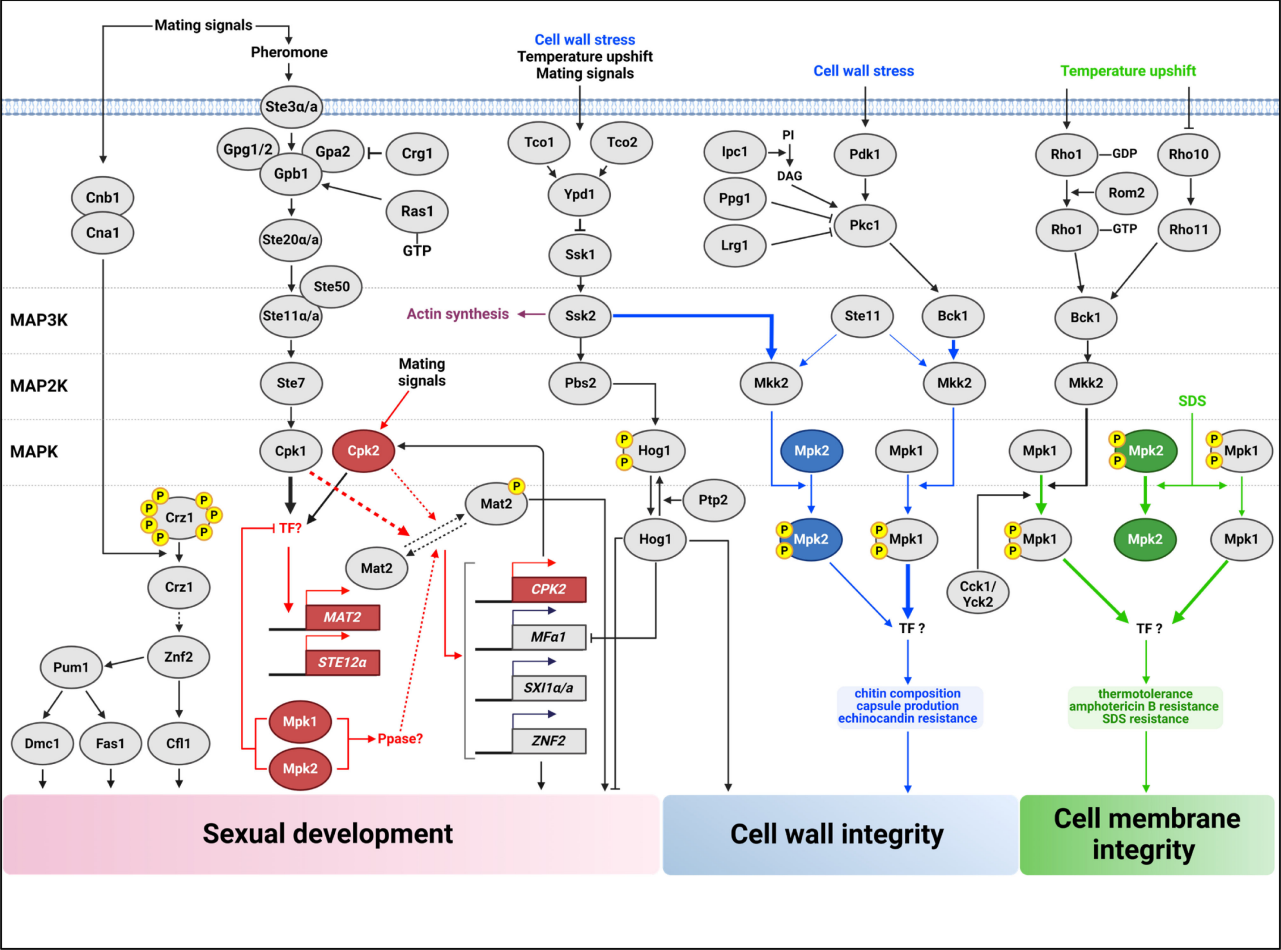
Schematic representation of the complex MAPK pathways for sexual reproduction and CWI of *C. neoformans*. In the left side, the pheromone-responsive Cpk1 MAPK pathway is known to activate Cpk1, which is believed to phosphorylate the transcription factor Mat2 for facilitating mating. While both Cpk1 and its paralog, Cpk2, contribute to this process, Cpk1 appears to play a predominant role. Once activated, Mat2 influences the expression of *MF*α*1*, *SXI1*α/*a*, and *ZNF2* and additionally modulates *CPK2* expression. Beyond this, Cpk1 appears to transcriptionally control Mat2 through an unknown transcription factor. Furthermore, Mpk1 and Mpk2 cooperate, probably targeting a phosphatase, which results in Mat2 inactivation. In the right side, the Mpk1 and Mpk2 MAPKs play redundant and distinct roles in maintaining cell wall and membrane integrity. This regulatory mechanism bifurcates into an Mpk2-dependent stress response—predominantly governed by Mpk1 but also influenced by Mpk2—and an Mpk2-independent stress response exclusively controlled by Mpk1. Aspects of CWI, such as chitin composition and capsule production, seem to be co-regulated by Mpk1 and Mpk2, with Mpk1 playing a major role in the modulation. Phosphorylation of Mpk2 is regulated by MAP2K Mkk2 like that of Mpk1. However, interestingly, Mpk2 phosphorylation is regulated by MAP3K Ssk2 and Ste11, not by Bck1. In contrast, cell membrane stability and thermotolerance are seemingly dictated solely by Mpk1. Under high-temperature stress, only Mpk1 was phosphorylated, while under SDS treatment, both Mpk1 and Mpk2 were dephosphorylated. Despite their differences in phosphorylation in response to types of membrane stress, phenotypic analysis revealed that only Mpk1 is involved in maintaining cell membrane integrity. Proteins and signaling pathways previously established in their roles are denoted by gray circles and black lines. In contrast, new contributions of this paper to understanding the mating process are highlighted in red, findings related to CWI are highlighted in blue, and those concerning cell membrane integrity are highlighted in green.

In pathogenic fungi, MAPK signaling cascades are critical for regulating essential cellular processes like development, stress response, and pathogenicity. Our study sheds light on the complex interactions among MAPKs of *C. neoformans*, focusing on the functional dynamics of Cpk2 and Mpk2 in conjunction with the primary MAPK pathways involving Cpk1 and Mpk1. We have shown that Cpk2 and Mpk2 possess functions that extend beyond mere redundancy to Cpk1 and Mpk1. These kinases are instrumental in compensating for mating defects in *cpk1*Δ mutants and cell wall integrity in *mpk1*Δ mutants via overexpression. Importantly, the dual deletion of Mpk1 and Mpk2 leading to the re-establishment of mating capabilities in *cpk1*Δ mutants underscores a sophisticated negative regulatory role that these kinases exert on the mating process. This finding highlights the complex interconnectivity between MAPK pathways, thus enriching our understanding of the fungal signaling network and its implication for development and pathogenicity (summarized in Fig. 7).

In our investigation of *C. neoformans*, we discovered that the minor MAPKs Cpk2 and Mpk2 exhibit structural and functional similarities to the major MAPKs Cpk1 and Mpk1. Contrary to *C. neoformans*, functional redundancy among MAPKs is observed but not a common feature in other fungi. For instance, most filamentous ascomycetes have a single CnCpk1 (*C. neoformans* Cpk1) ortholog. In *Magnaporthe oryzae*, the sole mating MAPK, Pmk1, is responsible for appressorium formation and invasive growth (28). Similarly, Fmk1 in *Fusarium oxysporum* is a crucial role for hyphal growth and virulence in plants yet non-essential in mammalian and invertebrate infection models, suggesting a host-specific role of MAPKs in virulence (29). In contrast, *Candida albicans* has two mating MAPKs, Cek1 and Cek2, which share functions in mating, biofilm formation, and cell wall damage response, with only Cek1 being essential in the yeast-to-hypha transition and virulence (30). *Ustilago maydis* also has two mating MAPKs, Kpp6 and its paralog Kpp2, with the former playing a more dominant role in appressorium penetration, while both MAPKs are involved in virulence and mating (31). In *C. neoformans*, while Cpk1 is an ortholog of Kpp2 and Cpk2 of Kpp6, the sexual differentiation defect is primarily observed in *cpk1*Δ mutants, unlike *U. maydis*. This indicates that in *C. neoformans*, Cpk2 does not independently drive the mating process but supplements Cpk1.

In this study, we found that during mating, *CPK2* expression is significantly upregulated, and its induction is contingent upon Cpk1, positioning Cpk2 as a potential downstream effector in the Cpk1-dependent pathway. Interestingly, while the absence of Cpk2 did not affect mating efficiency, its overexpression reinstated wild-type mating capabilities in *cpk1*Δ mutants. The necessity for Cpk1 to activate Cpk2 during mating in *C. neoformans* is not entirely clear. Nonetheless, the ability of overexpressed Cpk2 to substitute for Cpk1 hints at a scenario where *CPK2* might be highly overexpressed or regulated differentially in natural mating environments of *C. neoformans*, such as pigeon guano, decayed wood, or eucalyptus trees (32), possibly acting as a compensatory regulator for Cpk1. It is known that *C. neoformans* can induce mating under environmental conditions, and it has been reported that pigeon guano, in particular, can induce mating even better than the V8 medium (33). Additionally, in this study, just as Cpk2 was found to be overexpressed in *C. neoformans* H99 under mating condition with V8, the transcriptome results of *C. neoformans* JEC21 under the same condition also reported that the Cpk2 ortholog gene CNE00350 was overexpressed by about 200-fold at 24 h, providing more concrete evidence for the complementary function of Cpk2 to the mating MAPK Cpk1 (34). Further research is required to explore this hypothesis and the environmental factors that might influence *CPK2* expression.

Our findings support that Cpk1 initiates the upregulation of *MAT2*, a key transcription factor in mating regulation, which in turn appears to be involved in the induction of *CPK2* during the mating of *C. neoformans*. Intriguingly, the concurrent deletion of *MPK1* and *MPK2* reinstates *MAT2* induction to wild-type levels in the absence of Cpk1. These observations suggest the potential involvement of an as-yet-undiscovered transcription factor that may regulate *MAT2* induction. This hypothetical transcription factor could be subject to positive phosphorylation by Cpk1 and negative regulation by an Mpk1/Mpk2-dependent dephosphorylation process. There is also the possibility that Cpk1 could directly activate Mat2 through phosphorylation, an effect that might be antagonized by an Mpk1/Mpk2-associated phosphatase (Fig. 7). Currently, the direct regulation of Mat2 by phosphorylation through Cpk1 is not established. Drawing parallels with *S. cerevisiae*, where the Ste12 transcription factor—orthologous to CnMat2—is phosphorylated by Kss1/Fus1, which are orthologous to Cpk1 (35), provides a basis for these proposed mechanisms. Further research is necessary to test these hypotheses and fully elucidate the regulatory pathways involved.

The occurrence of two CWI MAPK isoforms is a distinctive feature of *C. neoformans*, not observed in other fungi. In *S. cerevisiae*, Slt2 is the singular CWI MAPK (3), while Pmk1 in *Schizosaccharomyces pombe* (36), Mkc1 in *C. albicans* (37), MpkA in *Aspergillus fumigatus* (38), and Mps1 in *M. oryzae* (39) each serve as the sole orthologs. To date, no additional CWI MAPK homologs have been reported in these fungal species. In *C. neoformans*, we found that Mpk2 can functionally assume some of Mpk1’s roles, notably in preserving cell wall integrity, indicating the evolutionary diversification of an additional CWI MAPK. Furthermore, our findings suggest that Mpk2 does not act merely as an auxiliary to Mpk1. Mpk1 and Mpk2 share roles in suppressing the Cpk1 MAPK pathway and maintaining cell wall integrity. In contrast, Mpk1 is critical for maintaining cell membrane integrity under stressed conditions such as high temperature, SDS exposure, and amphotericin B treatment, whereas Mpk2 is not. This difference likely stems from the distinct activation of the shared upstream Mkk2 MAP2K: Bck1 specifically activates Mpk1, while Ssk2 and Ste11 preferentially activate Mpk2. The interaction prediction between Ssk2 and Mkk2 using AlphaFold2-multimer suggests that their kinase domains can form multimers (Fig. S3C). This indicates that Ssk2 and Ste11, which are not MAP3Ks in the CWI pathway, might also interact with Mkk2. The roles of these highly similar MAPKs could be attributed to variations in when they are expressed or where they are located within the cell. This difference in timing and cellular positioning might be the underlying reason why these kinases, despite their similarities, participate in different cellular functions. These findings collectively imply a complex interplay among all MAPK signaling components in regulating the pathobiological traits of *C. neoformans*.

Our current and previous findings indicate that in *C. neoformans*, the pheromone-responsive MAPK and CWI MAPKs are commonly involved in maintaining cell wall integrity, a relationship that contrasts with their antagonistic roles during sexual reproduction. Previously, we demonstrated that the double deletion mutant *mpk1*Δ *cpk1*Δ exhibits greater sensitivity to cell wall stress than each single deletion mutant, suggesting a collaborative function of both MAPKs in the CWI pathway (40). In the present study, the additional removal of *CPK2* or *MPK2* did not further reduce cell wall stress resistance in the *mpk1*Δ *cpk1*Δ strain, suggesting that Cpk2 and Mpk2 have limited roles in these processes. In *C. albicans*, where the mating-related MAPKs, Cek1 and Cek2, are phosphorylated in response to cell wall stressors like tunicamycin and zymolyase, *CEK1* deletion results in more diminished growth under cell wall stress that *CEK2* deletion does (30). Furthermore, the deletion of *CEK1* or *CEK2* in an *mkc1*Δ background (an ortholog of CnMpk1) exacerbates its cell wall stress resistance, indicating a positive interaction between the mating MAPK and CWI MAPK pathways in this organism (30). Given that sexual reproduction involves the recognition and fusion of mating-type cells, filamentation, and spore formation, we proposed that the dynamic regulation of cell wall integrity is essential during these developmental stages. This could explain the observed interconnection between the pheromone-responsive MAPK and CWI MAPK pathways, as depicted in Fig. 7.

In summary, our study sheds light on the intricate interactions between the pheromone-responsive MAPK and CWI MAPK pathways, revealing the subtle yet significant roles of the lesser-studied MAPKs, Cpk2 and Mpk2. These findings underscore the MAPK paralog’s overlapping and distinct roles in stress response and environmental adaptation, underscoring a sophisticated level of evolutionary development that likely contributes to the pathogen’s versatility and tenacity across varied ecological and host settings. Our work lays the groundwork for future research aimed at an in-depth examination of each MAPK’s role. As we further decipher the molecular intricacies of MAPK signaling in *C. neoformans*, we advance toward a more nuanced understanding of the interplay between fungal pathogens and their host environments, which is paramount for a complete comprehension of fungal pathobiology.

## MATERIALS AND METHODS

### Illustration of a phylogenetic tree of fungal MAPKs

We constructed a phylogenetic tree using MAPK protein sequences from 19 representative fungi species. After searching the fungal genome database FungiDB (fungidb.org) and retrieving the protein sequences, we utilized the MEGA 11 to illustrate the phylogenetic tree (41). The evolutionary analysis was conducted using the maximum likelihood method and a model based on the Jones-Taylor-Thornton (JTT) matrix (42). The initial tree for heuristic search was automatically generated by employing the Neighbor-Join and BinNJ algorithms to create a matrix of pairwise distances, which were estimated utilizing the JTT model.

### Domain structural analysis of *C. neoformans* MAPKs: Cpk1, Cpk2, Mpk1, and Mpk2

Protein sequences of MAPKs Cpk1, Cpk2, Mpk1, and Mpk2 were obtained from FungiDB and utilized to identify kinase domains with InterPro (http://www.ebi.ac.uk/interpro/). For the structural prediction, AlphaFold2, an advanced machine-learning tool developed for the prediction of protein structures, was employed. AlphaFold2 was accessed via ColabFold, a user-friendly web-based interface that simplifies the utilization of AlphaFold2’s complex algorithms (19). Predicted protein MAPK domains were illustrated with MolStar from the RCSB protein database (https://www.rcsb.org/3d-view).

### Construction of the *C. neoformans* MAPK mutants

All combinations of MAPK deletion mutants of *C. neoformans* serotype A H99 strain as a parental strain were constructed. *cpk1*Δ *cpk2*Δ, *mpk1*Δ *mpk2*Δ, *mpk1*Δ *cpk2*Δ, *cpk1*Δ *mpk2*Δ, *cpk2*Δ *mpk2*Δ, *mpk1*Δ *cpk1*Δ *mpk2*Δ, *mpk1*Δ *cpk1*Δ *cpk2*Δ, *cpk1*Δ P*_H3_:CPK2*, *cpk1*Δ P*_H3_:CPK2 mat2*Δ, *mpk1*Δ P*_H3_:MPK2*, and *mat2*Δ (*MAT***a** and *MAT*α) were newly generated in this study (Table S1). Gene deletion cassettes were produced by split marker-based double joint PCR containing G418/neomycin resistance marker (*NEO*) or hygromycin B resistance marker (*HYG*). The 5′- and 3′-flanking regions of the target genes were amplified by PCR using wild-type genomic DNA as a template, with L1/L2 and R1/R2 primer pairs. The *NEO* and *HYG* markers were amplified by PCR with M13F and M13R primers from pNEO and pHYG plasmid. After PCR of 5′- and 3′-flanking regions and selection markers, the second-round PCR produced *NEO*-split and *HYG*-split gene disruption cassette with listed primer pairs: L1/NSM1 and R2/NSM2 for *NEO-*split cassettes and L1/HSM1 and R2/HMS2 for *HYG-*split cassettes, respectively (Table S2). The first-round PCR was performed with DyeMIX Tenuto (Enzynomics, Daejeon, South Korea), and the second round was performed with ExTaq polymerase (Takara Bio, Tokyo, Japan) (43). Gene disruption cassettes from PCR products were introduced by biolistic transformation. Each parental strain was cultured in 50 mL YPD (yeast extract-peptone-dextrose) medium for 16 h at 30°C 200 rpm. Cells were spun down, resuspended with 5 mL fresh YPD, spread onto YPD agar medium containing 1 M sorbitol, and further incubated for 3 h at 30°C. Gene disruption cassettes were mixed with 600 µg of 0.6 µm gold bead (Bio-Rad Laboratories, CA, USA), 2.5 M of calcium chloride, and 2 µL of spermidine (S-0266, Sigma Aldrich, MA, USA) and introduced into parental cells using a particle delivery system (PDS-1000, Bio-Rad Laboratories) at a pressure of 1,500 psi. After 4 h of incubation at 30°C, cells were scrapped and spread on YPD agar medium containing G418 (40 µg/mL) or hygromycin B (200 µg/mL). The genotypes of each screened mutant were confirmed with diagnostic PCR and Southern blot analyses.

### Gene expression analysis using quantitative RT-PCR and northern blotting

To monitor the mRNA abundance of target genes, quantitative reverse transcription-PCR (qRT-PCR) and northern blotting were performed. For RNA extraction, cells were cultivated overnight at 30°C in 50 mL of YPD broth. To assess gene expression associated with mating processes, the cultured cells were centrifuged at 3,000 rpm for 5 min and washed thrice with phosphate-buffered saline (PBS). A combined cell mixture, consisting of equal concentrations (10^8^ cells/mL) of *MAT***a** and *MAT*α cells, was then prepared. These cells were plated on V8 media and incubated for 16 h at room temperature in the dark. After incubation, the cells were collected and subjected to overnight lyophilization (44).

In a separate procedure to evaluate gene expression related to the CWI pathway, the wild-type and mutant strains were centrifuged at 3,000 rpm for 5 min. The supernatant was discarded, and the cell pellet was resuspended in 50 mL of fresh YPD broth. Each cell was subcultured in three flasks until OD_600_ reached 0.8, at which point cells were treated with 1% CFW. Subsequently, the samples were spun down and lyophilized overnight. Lyophilized samples were powdered with a 3 mm glass bead, and total RNA was extracted using a commercial RNA kit (iNtRON Biotechnology, Seongnam, South Korea). The RNA concentration was measured by Nanodrop (Bio-Rad Laboratories), and 4,000 ng of total RNA was synthesized using reverse transcriptase (ThermoFisher Scientific, MA, USA). qRT-PCR was performed (CFX96, Bio-Rad Laboratories) with target gene primer using TB Green (ThermoFisher Scientific). The relative gene expression was analyzed with the 2^−ΔΔCT^ method (45), and the data were illustrated with Prism 8.0. For the northern blotting analysis, 10 µg of total RNA was used to prepare the membrane. Radioactively labeled probes, synthesized with gene-specific primers for mating pheromone *MFα1* and actin *ACT1* according to previously described methods, were utilized for membrane hybridization (35).

### Mating filament production and sporulation assay

To evaluate unilateral and bilateral mating efficiencies, MAPK mutants constructed in the *MAT*α H99 strain and *MAT***a** YL99**a** background were individually cultured in YPD medium at 30°C for 16 h and washed twice with PBS. For unilateral mating, *MAT*α wild-type and MAPK mutant cells were mixed at a concentration of 10^7^ cells/mL with *MAT***a** wild-type cells. Bilateral mating was conducted by combining mutant cells of one mating type at the same concentration with the analogous mutants of the opposite mating type. The mixtures were then spotted onto V8 mating medium (pH 7.0) or MS medium (pH 5.8) and incubated at 25°C in the dark for a period of 7–30 days. Filamentous growth and sporulation were observed and documented using a differential interference contrast microscope ECLIPSE Ni (Nikon, Tokyo, Japan) equipped with a DS-Qi2 camera (Nikon) and a BX51 microscope (Olympus, Tokyo, Japan) equipped with a SPOT Insight digital camera (Diagnostic Instruments, Inc., MI, USA) (44).

### Phenotypic analysis of the *C. neoformans* MAPK mutants

To analyze the phenotypic change of MAPK mutants, a spotting assay was performed. The wild-type and MAPK mutants were grown overnight at 30°C in YPD broth, serially diluted 10-fold (1–10^−4^ dilutions), and spotted on YPD agar plates containing various concentrations stress agents: antifungal agents (anidulafungin, micafungin, and amphotericin B), cell wall stressors (Congo red and calcofluor white), and cell membrane stressor (SDS).

### Protein extraction and immunoblotting assay

The wild-type (H99), *mpk1*Δ, *mpk2*Δ, *mpk1*Δ *mpk2*Δ, *bck1*Δ, *mkk2*Δ, *ssk2*Δ, and *ste11*Δ strains were grown overnight in YPD at 30°C. Cells were synchronized to OD_600_ = 0.2 with fresh YPD medium and further incubated at 30°C. After the cells reach OD_600_= 0.8, temperature upshift from 30°C to 39°C or cell wall disturbing agent 1% CFW added. Samples were spun down and frozen in liquid nitrogen. Frozen samples were resuspended with 0.7 mL of cell lysis buffer (50 mM Tris [pH 7.5], 1% sodium deoxycholate, 5 mM sodium pyrophosphate, 0.1 µM sodium orthovanadate, 50 mM NaF, 0.1% SDS, 1% Triton X-100, 0.5 µM phenylmethylsulfonyl fluoride [PMSF], and 1× protease inhibitor cocktail solution [protease inhibitor cocktail set IV, Calbiochem]) and subjected to bead-beating. Then, the samples were centrifuged for 20 min at 13,000 rpm 4°C. Supernatants were moved to new e-tubes and quantified with Pierce BCA protein assay kit (ThermoFisher Scientific). Bovine serum albumin standard protein (ThermoFisher Scientific) and extracted protein samples were reacted with BCA solution and incubated at 37°C for 30 min. After quantification, the protein concentration was equalized, and the 5× sample buffer (250 mM Tris-HCl [pH 6.8], 50% glycerol, 10% SDS, and 0.5% bromophenol blue) and 2-mercaptoethanol (final 5%) were added. Samples were heated to 95°C for 15 min and stored at −20°C for immunoblotting. First antibodies were used as follows: phospho-p44/42 MAPK antibody (Cell signaling, #4370, 2,000:1), anti β-actin antibody (sc-47778, 1,000:1), and anti α-tubulin antibody (sc-8035, 1,000:1). Second antibodies were used as follows: mouse anti-rabbit IgG-horseradish peroxidase (HRP; sc-2357, 4,000:1) for detecting phospho-Mpk1, m-IgG Fc BP-HRP for detecting β-actin (sc-525409, 2,000:1), and m-IgGκ BP-HRP for detecting α-tubulin (sc-516102, 2,000:1). All antibodies except phospho-p44/42 MAPK antibody is a product of Santa Cruz Biotechnology, TX, USA. HRP was detected by chemiluminescence using the enhanced chemiluminescence (ECL) system (D-Plus ECL Pico Alpha System), and images of the western blot membrane were pictured with ChemiDoc XRS+ (Bio-Rad Laboratories).

### Quantitative measurement of chitin content in the *C. neoformans*

To assess chitin content in both wild-type and MAPK mutant strains, cells were incubated overnight in a shaking environment at 30°C using 2 mL of YPD medium. These cells were synchronized with OD_600_ 0.2 and further incubated 24 h at 30°C. Then the samples were collected, washed twice with pH 7.5 PBS, and reintroduced into a PBS solution. A subsequent staining process involved the use of 25 µg/mL CFW for a duration of 30 min at room-temperature in the dark. After being washed twice with PBS, the stained cells were resuspended in PBS, and their images were captured using fluorescence microscopy ECLIPSE Ni (Nikon). The fluorescence indicative of chitin content was quantitatively analyzed in at least 50 individual cells using ImageJ/Fiji software, following previously established methods (46).

### Capsule production assay

During the cryptococcal capsule induction assay, individual strains were cultured in a liquid YPD medium at 30°C for a duration of 16 h. Subsequently, the cells were washed with PBS, and 3 µL aliquots were applied onto Littman’s agar medium (LIT), a process well-documented in previous studies (47, 48). Then the LIT plates were incubated for an additional 2 days at 37°C. Post-incubation, India ink (Remel Inc., CA, USA) was employed to stain and visualize the capsules, which were subsequently examined under a microscope. The quantification of the capsule sizes involved measuring the diameters of both the capsules and cells microscopically, with the aid of Nikon NIS software, for enhanced accuracy (49).

## Data Availability

All data are presented in this paper or available in the supplemental materials. All strains and materials utilized in this study are available upon request.

## SUPPLEMENTAL MATERIAL

Fig. S1 mbio.01156-24-s0001.pdf Construction of mutant strains was established through Southern blot analysis.

Fig. S2 mbio.01156-24-s0002.pdf Cell wall stress phosphorylates Mpk1 and Mpk2, with Mpk2's overexpression able to partially compensate for *mpk1*Δ.

Fig. S3 mbio.01156-24-s0003.pdf Mpk2 functions similarly to, yet is distinctly regulated from, Mpk1 through its own phosphorylation controls.

Fig. S4 mbio.01156-24-s0004.pdf The MAPKs are intricately involved in mating, regulating the Cpk1 MAPK pathway.

Table S1 mbio.01156-24-s0005.pdf List of strains used in this study.

Table S2 mbio.01156-24-s0006.pdf List of primers used in this study.

ASM does not own the copyrights to Supplemental Material that may be linked to, or accessed through, an article. The authors have granted ASM a non-exclusive, world-wide license to publish the Supplemental Material files. Please contact the corresponding author directly for reuse.
