# Supplementary material for: Unraveling the cryptic functions of mitogen-activated protein kinases Cpk2 and Mpk2 in Cryptococcus neoformans
Source: mBio. 2024 Jun 14;15(7):e01156-24. doi: 10.1128/mbio.01156-24 (PMC11253600; doi:10.1128/mbio.01156-24)
Supplement: Fig. S1 — Construction of mutant strains was established through Southern blot analysis. [file mbio.01156-24-s0001.pdf]

Jang et al. (Supplementary Figure 1)

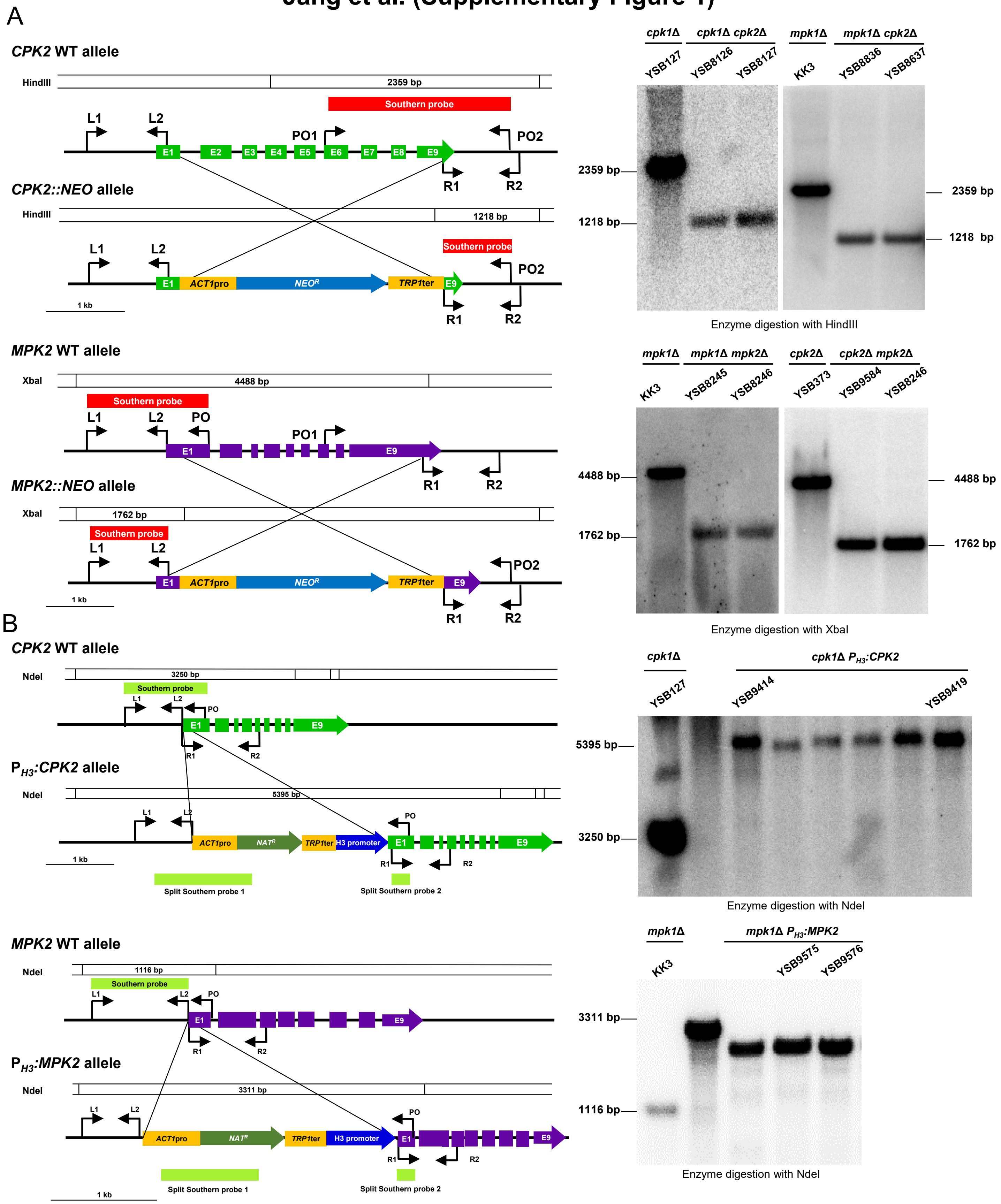

(continued)

Jang et al. (Supplementary Figure 1)

C

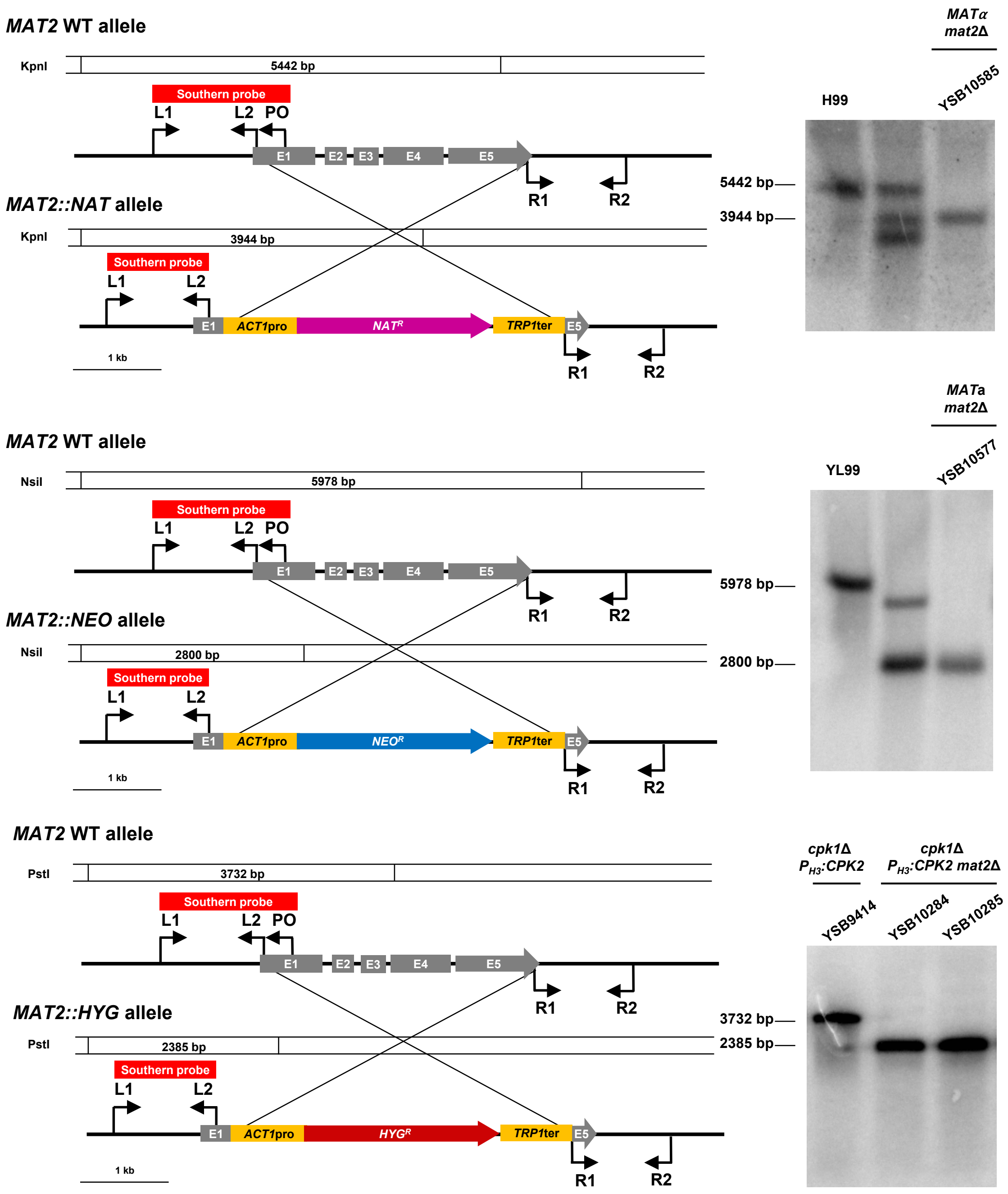

(continued)

Jang et al. (Supplementary Figure 1)

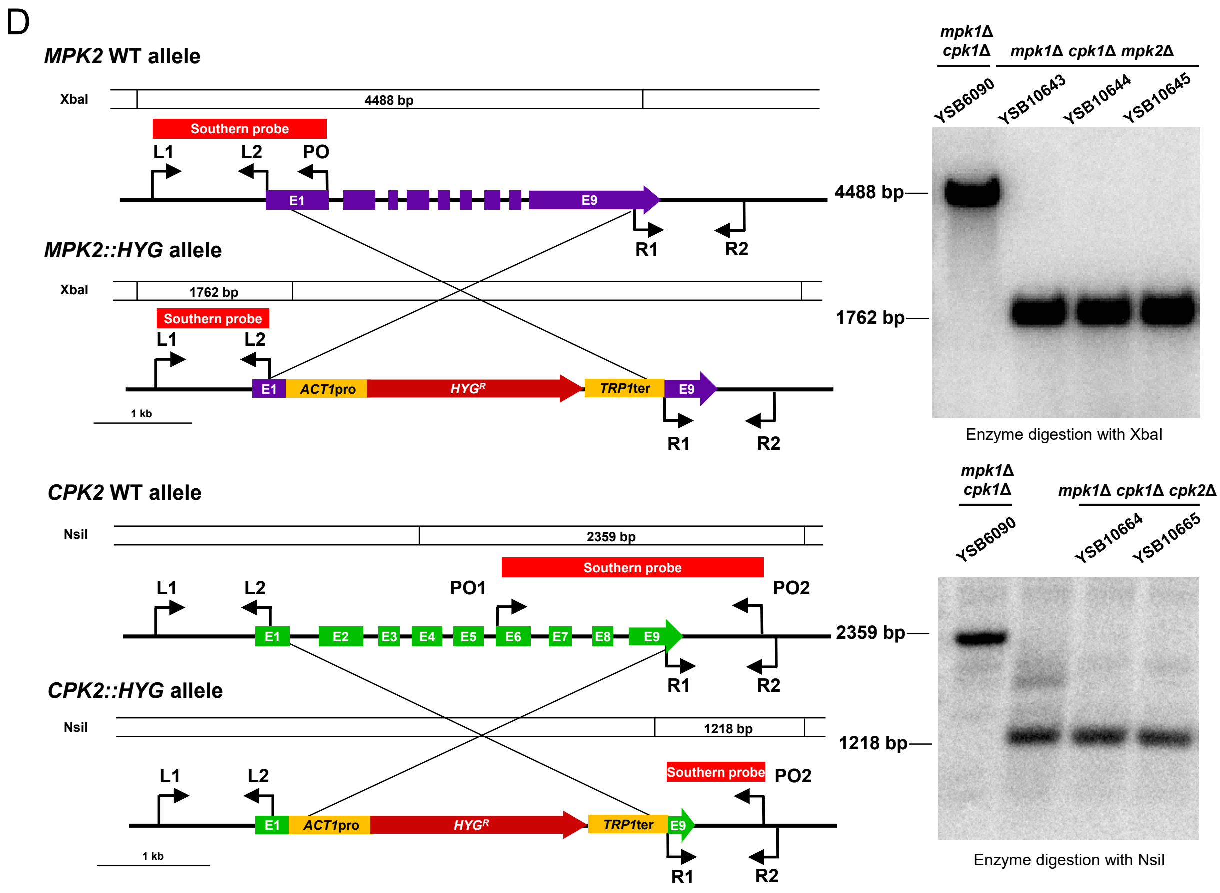

Figure S1. Construction of mutant strains were established through Southern blot analysis. The target MAPK genes were replaced with neomycin or hygromycin resistance genes (*NEO*^{R} or *HYG*^{R}), and the genes were delivered using the gene gun biolistic transformation method (see Material and Method). Graphical representations depict the strategies for homologous recombination between the wild-type gene and the deletion cassette. The newly constructed strains were verified through diagnostic PCR and further validated by Southern blot using dCTP labeled with the radioactive phosphorus P^{32}. The primer sets used for these procedures are detailed in Table S2.
