# Supplementary material for: Unraveling the cryptic functions of mitogen-activated protein kinases Cpk2 and Mpk2 in Cryptococcus neoformans
Source: mBio. 2024 Jun 14;15(7):e01156-24. doi: 10.1128/mbio.01156-24 (PMC11253600; doi:10.1128/mbio.01156-24)
Supplement: Fig. S2 — Cell wall stress phosphorylates Mpk1 and Mpk2, with Mpk2's overexpression able to partially compensate for mpk1Δ. [file mbio.01156-24-s0002.pdf]

Jang et al. (Supplementary Figure 2)

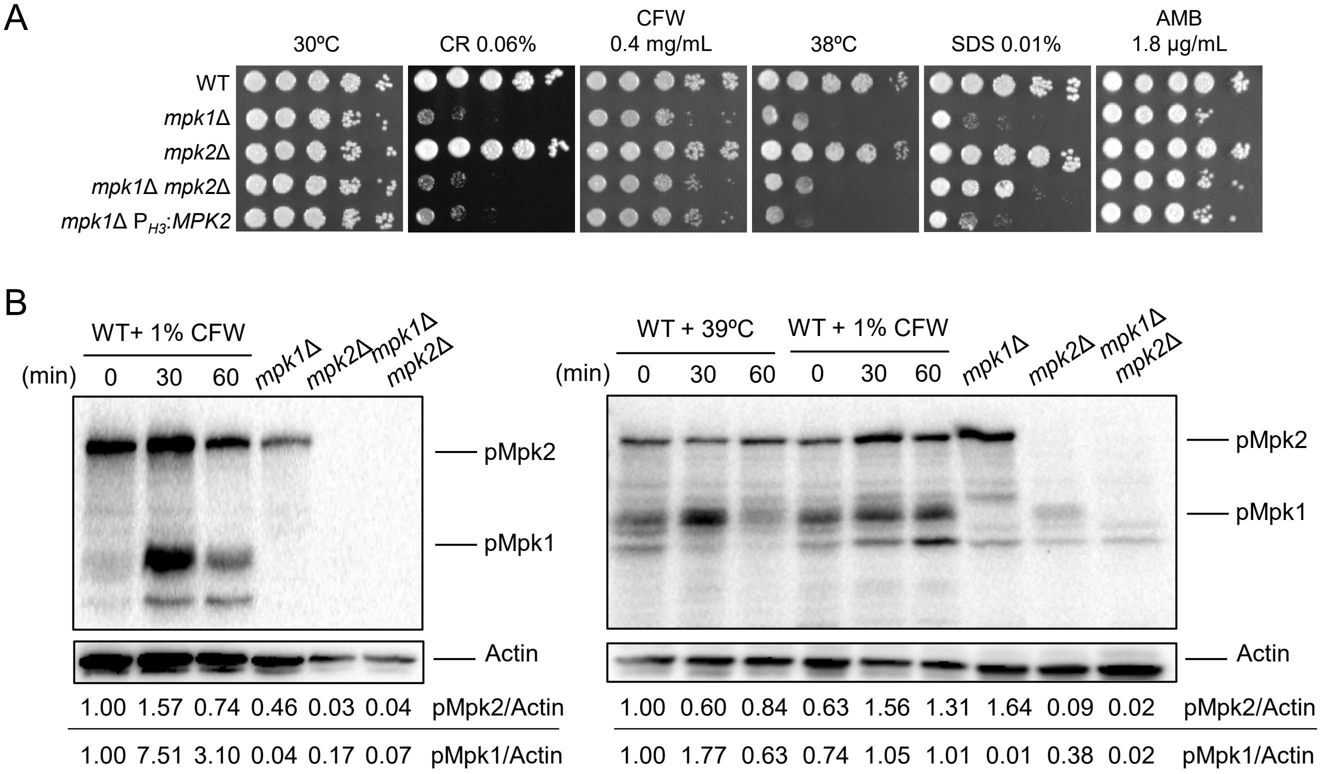

Figure S2. Cell wall stress phosphorylates Mpk1 and Mpk2, with Mpk2's overexpression able to partially compensate for *mpk1*Δ. A) Wild-type, *mpk1*Δ, *mpk2*Δ, *mpk1*Δ *mpk2*Δ (YSB8246), and *mpk1*Δ P_{H3}:*MPK2* (YSB9576) were cultured and added 0.06% CR, 0.4 mg/mL CFW, 5 ug/mL FCZ, at 38°C, 0.01% SDS, and 1.8 ug/mL AMB, then repeated the spotting experiments. The used *mpk1*Δ *mpk2*Δ and *mpk1*Δ P_{H3}:*MPK2* are independent additional strains from Fig. 3C. (B) After exposing wild-type strains to 39ºC or 1% CFW for 0, 30, and 60 minutes, the phosphorylation of Mpk1 and Mpk2 was assessed. Phosphorylation of both Mpk1 and Mpk2 was detected simultaneously using an anti phospho-p42/44 antibody (Cell Signaling, #4370), and *mpk1*Δ, *mpk2*Δ, and *mpk1*Δ *mpk2*Δ were used as controls to confirm the identity of each protein band. Actin was used as the loading control.
