# Supplementary material for: Unraveling the cryptic functions of mitogen-activated protein kinases Cpk2 and Mpk2 in Cryptococcus neoformans
Source: mBio. 2024 Jun 14;15(7):e01156-24. doi: 10.1128/mbio.01156-24 (PMC11253600; doi:10.1128/mbio.01156-24)
Supplement: Fig. S3 — Mpk2 functions similarly to, yet is distinctly regulated from, Mpk1 through its own phosphorylation controls. [file mbio.01156-24-s0003.pdf]

Jang et al. (Supplementary Figure 3)

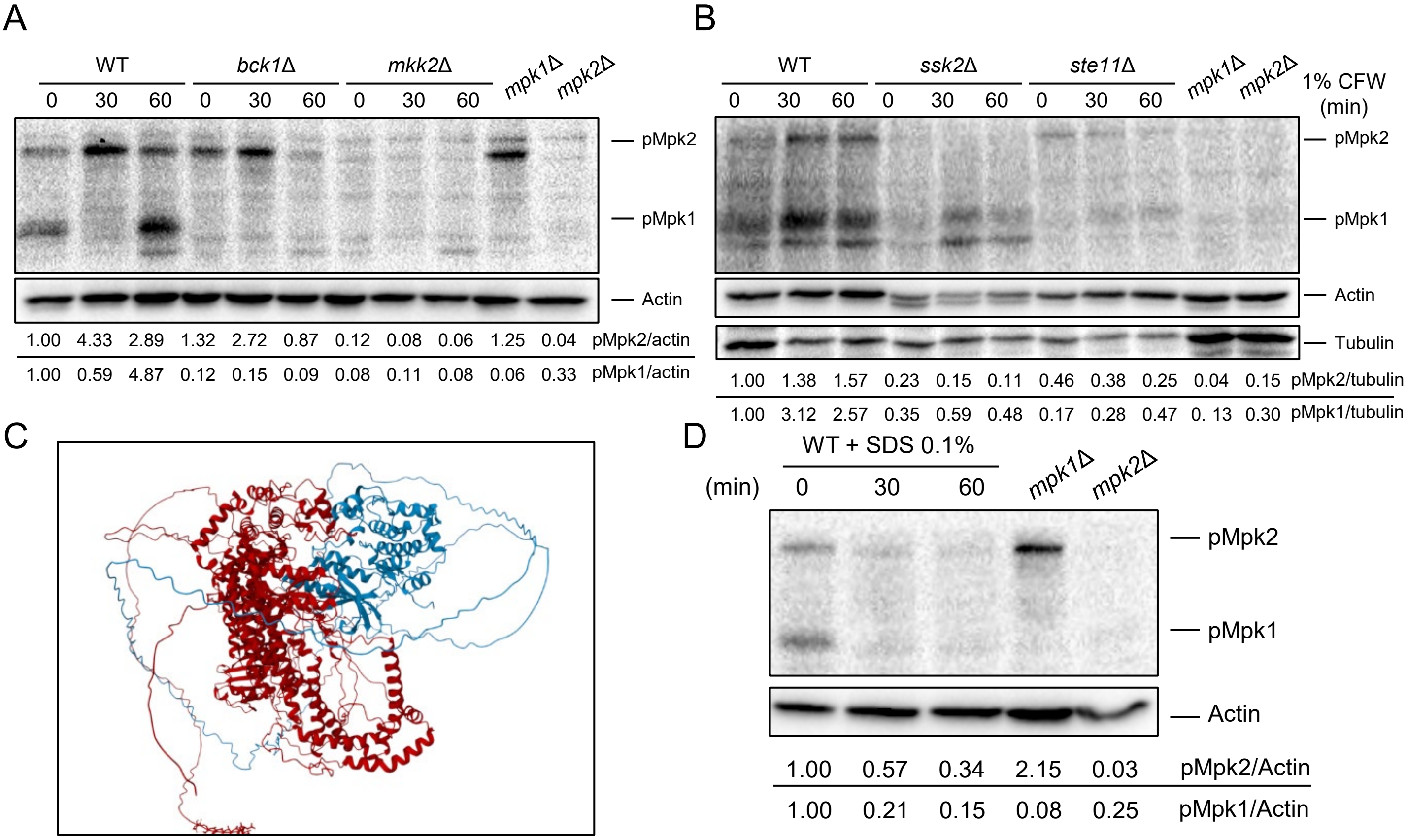

Figure S3. Mpk2 functions similarly to, yet is distinctly regulated from, Mpk1 through its own phosphorylation controls. (A-B) In wild-type, *mpk1*Δ *mpk2*Δ, *bck1*Δ, and *mkk2*Δ, *ssk2*Δ, and *ste11*Δ the phosphorylation levels of Mpk1 and Mpk2 were measured by western blot after exposure to 1% CFW. These measurements were carried out as biological replicates, following the initial results presented in Figure 4A, 4B. (C) To predict the interaction between Ssk2 (red) and Mkk2 (blue), their structures were forecasted using AlphaFold2. The kinase domains of the two kinases are anticipated to be proximal, suggesting a potential interaction. (D) After treating wild-type with 0.1% SDS, the phosphorylation of Mpk1 and Mpk2 were measured. Similar to what is observed in Figure 5C, dephosphorylation of Mpk1 and Mpk2 induced by SDS was noted.
