# Supplementary material for: Unraveling the cryptic functions of mitogen-activated protein kinases Cpk2 and Mpk2 in Cryptococcus neoformans
Source: mBio. 2024 Jun 14;15(7):e01156-24. doi: 10.1128/mbio.01156-24 (PMC11253600; doi:10.1128/mbio.01156-24)
Supplement: Fig. S4 — The MAPKs are intricately involved in mating, regulating the Cpk1 MAPK pathway. [file mbio.01156-24-s0004.pdf]

Jang et al. (Supplementary Figure 4)

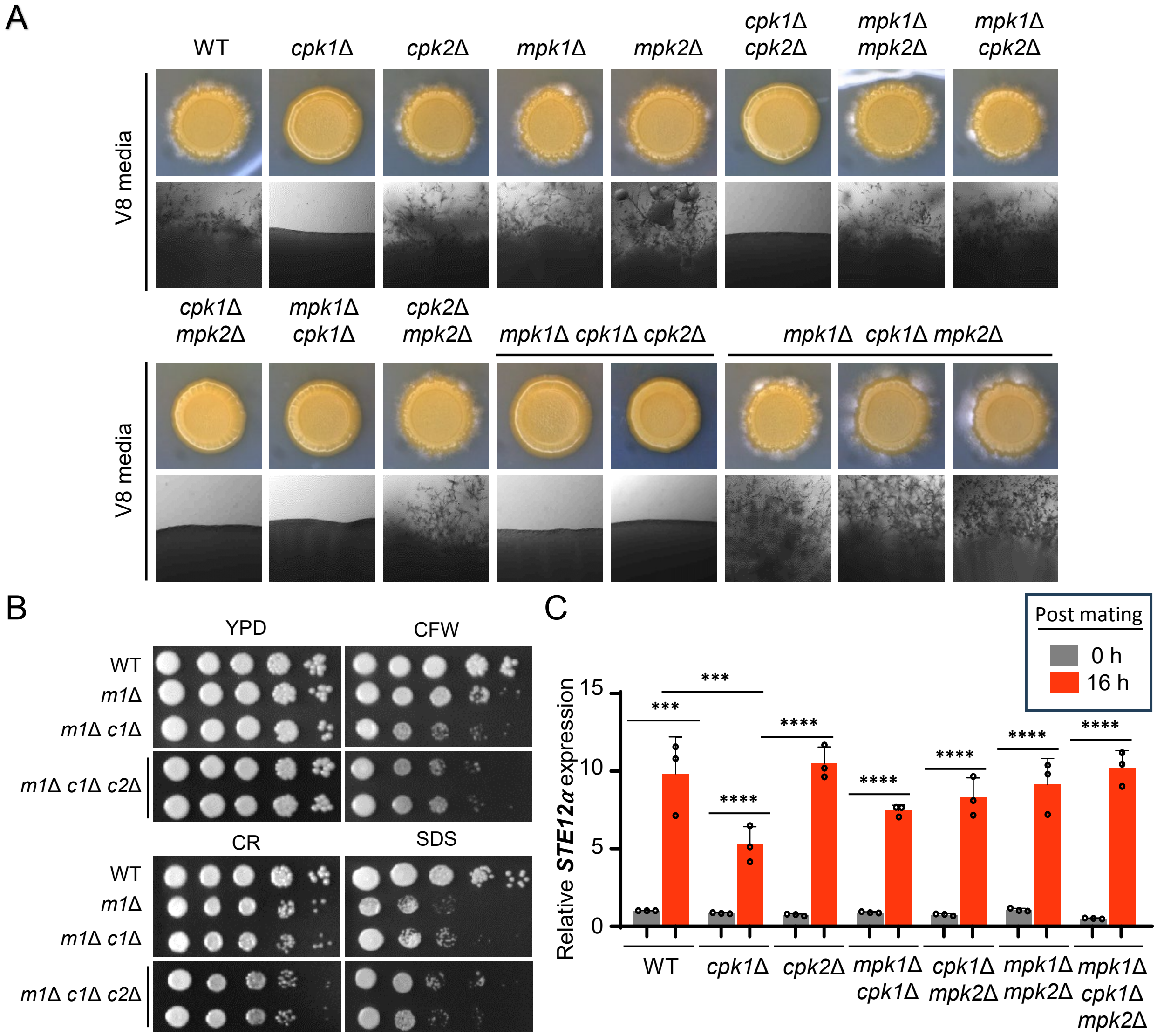

Figure. S4. The MAPKs are intricately involved in mating, regulating the Cpk1 MAPK pathway. (A) The filamentation of the MAPK mutants presented above was reconfirmed in V8 media. For *mpk1*Δ *cpk1*Δ *cpk2*Δ, two independent strains were used, and for *mpk1*Δ *cpk1*Δ *mpk2*Δ, three independent strains were utilized, all yielding consistent results. (B) Wild-type, *mpk1*Δ, *mpk1*Δ *cpk1*Δ, and two independent *mpk1*Δ *cpk1*Δ *cpk2*Δ strains were subjected to spot assays under cell wall and cell membrane stress conditions of 0.05% CR, 0.5 mg/ml CFW, and 0.01% SDS. (C) Under the same conditions as in figures C and E, the expression levels of *STE12α* in the wild-type and each MAPK mutant were measured. The statistical importance of the results was ascertained by conducting a one-way ANOVA followed by Tukey's test for multiple comparisons. The levels of significance were denoted as follows: '***' for *P* values less than 0.001, '****' for less than 0.0001, and 'NS' indicating a lack of statistical significance.
