# Supplementary material for: Unraveling the cryptic functions of mitogen-activated protein kinases Cpk2 and Mpk2 in Cryptococcus neoformans
Source: mBio. 2024 Jun 14;15(7):e01156-24. doi: 10.1128/mbio.01156-24 (PMC11253600; doi:10.1128/mbio.01156-24)
Supplement: Table S1 — List of strains used in this study. [file mbio.01156-24-s0005.pdf]

Supplementary Table 1. List of strains used in this study.

Strain	Genotype	Parent	Reference
H99	*MAT*α		(1)
YL99	*MAT***a**		(2)
YSB127	*MAT*α *cpk1*Δ::*NAT*-STM #*184*	H99	(3)
YSB373	*MAT*α *cpk2*Δ::*NAT*-STM #*122*	H99	(3)
YSB264	*MAT*α *ssk2*Δ::*NAT*-STM #*210*	H99	(4)
YSB273	*MAT*α *bck1*Δ::*NAT*-STM #*43*	H99	(5)
YSB313	*MAT*α *ste11*Δ::*NAT*-STM #*242*	H99	(4)
YSB330	*MAT*α *mkk2*Δ::*NAT*-STM #*224*	H99	(5)
YSB8126	*MAT*α *cpk1*Δ::*NAT cpk2*Δ::*NEO*	YSB127	This study
YSB8127	*MAT*α *cpk1*Δ::*NAT cpk2*Δ::*NEO*	YSB127	This study
YSB9414	*MAT*α *cpk1*Δ::*NAT* P_{H3}:*CPK2*::*NEO*	YSB127	This study
YSB9419	*MAT*α *cpk1*Δ::*NAT* P_{H3}:*CPK2*::*NEO*	YSB127	This study
KK3	*MAT*α *mpk1*Δ::*NAT* -STM #*150*	H99	(5)
YSB3236	*MAT*α *mpk2*Δ::*NAT* -STM #*102*	H99	(3)
YSB8245	*MAT*α *mpk1*Δ::*NAT mpk2*Δ::*NEO*	KK3	This study
YSB8246	*MAT*α *mpk1*Δ::*NAT mpk2*Δ::*NEO*	KK3	This study
YSB9575	*MAT*α *mpk1*Δ::*NAT* P_{H3}:*MPK2*::*NEO*	KK3	This study
YSB9576	*MAT*α *mpk1*Δ::*NAT* P_{H3}:*MPK2*::*NEO*	KK3	This study
YSB8636	*MAT*α *mpk1*Δ::*NAT cpk2*Δ::*NEO*	KK3	This study
YSB8637	*MAT*α *mpk1*Δ::*NAT cpk2*Δ::*NEO*	KK3	This study
YSB9206	*MAT*α *cpk1*Δ::*NAT mpk2*Δ::*NEO*	YSB127	This study
YSB9207	*MAT*α *cpk1*Δ::*NAT mpk2*Δ::*NEO*	YSB127	This study
YSB9584	*MAT*α *cpk2*Δ::*NAT mpk2*Δ::*NEO*	YSB373	This study
YSB9585	*MAT*α *cpk2*Δ::*NAT mpk2*Δ::*NEO*	YSB373	This study
YSB6090	*MAT*α *mpk1*Δ::*NAT cpk1*Δ::*NEO*	YSB3814	(6)
YSB10643	*MAT*α *mpk1*Δ::*NAT cpk1*Δ::*NEO mpk2*Δ::*HYG*	YSB6090	This study
YSB10644	*MAT*α *mpk1*Δ::*NAT cpk1*Δ::*NEO mpk2*Δ::*HYG*	YSB6090	This study
YSB10645	*MAT*α *mpk1*Δ::*NAT cpk1*Δ::*NEO mpk2*Δ::*HYG*	YSB6090	This study
YSB10664	*MAT*α *mpk1*Δ::*NAT cpk1*Δ::*NEO cpk2*Δ::*HYG*	YSB6090	This study
YSB10665	*MAT*α *mpk1*Δ::*NAT cpk1*Δ::*NEO cpk2*Δ::*HYG*	YSB6090	This study
YSB10577	*MAT***a** *mat2*Δ::*NEO*	YL99	This study
YSB10585	*MAT*α *mat2*Δ::*NAT*-STM #*210*	H99	This study
YSB10284	*MAT*α *cpk1*Δ::*NAT* P_{H3}:*CPK2*::*NEO mat2*Δ::*HYG*	YSB9414	This study
YSB10285	*MAT*α *cpk1*Δ::*NAT* P_{H3}:*CPK2*::*NEO mat2*Δ::*HYG*	YSB9414	This study

1. Perfect JR, Ketabchi N, Cox GM, Ingram CW, Beiser CL. 1993. Karyotyping of *Cryptococcus neoformans* as an epidemiological tool. J Clin Microbiol 31:3305-9.
2. Semighini CP, Averette AF, Perfect JR, Heitman J. 2011. Deletion of *Cryptococcus neoformans* AIF ortholog promotes chromosome aneuploidy and fluconazole-resistance in a metacaspase-independent manner. PLoS Pathog 7:e1002364.
3. Lee KT, So YS, Yang DH, Jung KW, Choi J, Lee DG, Kwon H, Jang J, Wang LL, Cha S, Meyers GL, Jeong E, Jin JH, Lee Y, Hong J, Bang S, Ji JH, Park G, Byun HJ, Park SW, Park YM, Adedoyin G, Kim T, Averette AF, Choi JS, Heitman J, Cheong E, Lee YH, Bahn YS. 2016. Systematic functional analysis of kinases in the fungal pathogen *Cryptococcus neoformans*. Nat Commun 7:12766.
4. Bahn YS, Geunes-Boyer S, Heitman J. 2007. Ssk2 mitogen-activated protein kinase kinase kinase governs divergent patterns of the stress-activated Hog1 signaling pathway in *Cryptococcus neoformans*. Eukaryot Cell 6:2278-89.
5. Kojima K, Bahn YS, Heitman J. 2006. Calcineurin, Mpk1 and Hog1 MAPK pathways independently control fludioxonil antifungal sensitivity in *Cryptococcus neoformans*. Microbiology (Reading) 152:591-604.
6. So YS, Jang J, Park G, Xu J, Olszewski MA, Bahn YS. 2018. Sho1 and Msb2 Play Complementary but Distinct Roles in Stress Responses, Sexual Differentiation, and Pathogenicity of *Cryptococcus neoformans*. Front Microbiol 9:2958.
