# Supplementary material for: Unraveling the cryptic functions of mitogen-activated protein kinases Cpk2 and Mpk2 in Cryptococcus neoformans
Source: mBio. 2024 Jun 14;15(7):e01156-24. doi: 10.1128/mbio.01156-24 (PMC11253600; doi:10.1128/mbio.01156-24)
Supplement: Table S2 — List of primers used in this study. [file mbio.01156-24-s0006.pdf]

Supplementary Table 2. List of primers used in this study.

Primer Name	Sequence (5' to 3')	Comments
**B79**	TGTGGATGCTGGCGGAGGATA	Screening primer
**B1026**	GTAAAACGACGGCCAGTGAAT	M13 forward universal primer
**B1027**	CAGGAAACAGCTATGACCATG	M13 reverse universal primer
**B1454**	AAGGTGTTCCCCGACGACGAATCG	*NAT* PCR primer SM1
**B1455**	AACTCCGTCGCGAGCCCCATCAAC	*NAT* PCR primer SM2
**B1886**	TGGAAGAGATGGATGTGC	*NEO* PCR primer SM1
**B1887**	ATTGTCTGTTGTGCCCAG	*NEO* PCR primer SM2
**B5751**	CGAAGAATCTCGTGCTTTC	*HYG* PCR primer SM1
**B5752**	ATTGACCGATTCCTTGCG	*HYG* PCR primer SM2
**B20938**	CGTTGTAGACATGTTGGTATGCT	*CPK1* knockout primer L1
**B20939**	TCACTGGCCGTCGTTTTACGTCATGCTGCCA CTTTGCTA	*CPK1* knockout primer L2
**B20940**	CATGGTCATAGCTGTTTCCTGCCAGTACCCA CCATCCTCTC	*CPK1* knockout primer R1
**B20941**	GATGGTGGGGTAGGAAGGAC	*CPK1* knockout primer R2
**B20942**	CCAGATTGTCCAGCTGCAAG	*CPK1* screening primer SO
**B20943**	TGGTATGGTCAAAAGGCGTG	*CPK1* Southern blot probe primer PO
**B20944**	CTGGTACGCATCTTCCCTCT	*CPK1* internal screening primer iLP
**B20945**	GGAATGCTGCTCACCGTTAG	*CPK1* internal screening primer iRP
**B8557**	CTGCGATTTCGGTCTTGCC	*CPK1* qRT primer qLP
**B8558**	GATACCACCTTGTAGCGAC	*CPK1* qRT primer qRP
**JOHE12031**	ATGTGCTTGGTTTGCCCGAG	*CPK2* knockout primer L1
**JOHE12032**	CTGGCCGTCGTTTTACAACCTGACTTTGCGA GGAGC	*CPK2* knockout primer L2
**JOHE12033**	GTCATAGCTGTTTCCTGGGAAGAGTTGAAG AGGCTG	*CPK2* knockout primer R1
**JOHE12034**	ACTGTGGCTGTTGTTCAGGC	*CPK2* knockout primer R2
**JOHE12035**	CCAAGGGAAGTCTACCAATAC	*CPK2* screening primer SO
**JOHE12037**	GGGGAAAGATTAGTGCGTC	*CPK2* Southern blot probe primer PO1
**JOHE12038**	GTGCGTAGATGAACGAGTG	*CPK2* Southern blot probe primer PO2
**B18149**	CGGAGGAGGGTGTTAGTGAG	*CPK2* internal screening primer iLP
**B18150**	GCGAGTTGGTTGTGGCTAAA	*CPK2* internal screening primer iRP
**B8555**	CCATCAGGTAGCAAAGTAGC	*CPK2* qRT primer qLP
**B8556**	CAAAGTACTTCAACAGCTT	*CPK2* qRT primer qRP
**B14551**	GATGGAGCAGAGGCGTGTAG	*CPK2* overexpression PCR primer L1

**B14552**	CACTCGAATCCTGCATGCGATAGGATGGGA AACGGA	*CPK2* overexpression PCR primer L2
**B14553**	CCACAACACATCTATCACATGTCAACGTCC ACTTGC	*CPK2* overexpression PCR primer R1
**B14554**	ACGCGATGAAGGTCTGTTTC	*CPK2* overexpression PCR primer R2
**B14555**	GATGGGTTTGACTGCTGGTT	*CPK2* overexpression screening primer SO
**B14556**	ATGTGGAAAAGGTGGGACAG	*CPK2* overexpression Southern blot probe primer PO
**B2470**	ATGGCAGCAAGCGTAACTC	*MPK2* knockout PCR primer L1
**B2471**	TCACTGGCCGTCGTTTTACGTTTTATGCCCG TTGTGTTG	*MPK2* knockout PCR primer L2
**B2472**	CATGGTCATAGCTGTTTCCTGCCCAAAGTCA GTCTGGTAACC	*MPK2* knockout PCR primer R1
**B2473**	ATACATCTTCGTAGCCCCG	*MPK2* knockout PCR primer R2
**B2474**	TCCAAATAGACCAAGCCC	*MPK2* screening primer SO
**B2475**	CGTTGAGTGTTTGGTAGCC	*MPK2* Southern blot probe primer PO
**B18151**	AGACGGGAGCGAGTAAGATG	*MPK2* internal primer iLP
**B18152**	CAGCAACTCGGCCAGAATAC	*MPK2* internal primer iRP
**B9412**	GACAGCAAATCCCTACACG	*MPK2* qRT primer qLP
**B15176**	CGTACGAGGAGCGGAACC	*MPK2* qRT primer qLP
**B14557**	GTGGAAGTGGACGACTCAGG	*MPK2* overexpression PCR primer L1
**B14558**	CACTCGAATCCTGCATGCTTCCGTAGAGTG GGACAA	*MPK2* overexpression PCR primer L2
**B14559**	CCACAACACATCTATCACATGACGAGCCCT TCAACT	*MPK2* overexpression PCR primer R1
**B14560**	TCTCTCTCAACGCCCTTCTC	*MPK2* overexpression PCR primer R2
**B14561**	TGTTGAAGAAGACGGTCGTG	*MPK2* overexpression PCR primer SO
**B14228**	TGAAGCATGATGAGCCTGTC	*MAT2* knockout PCR primer L1
**B14229**	TCACTGGCCGTCGTTTTACAGGGGTGAGAC ATCAAATCG	*MAT2* knockout PCR primer L2
**B14230**	CATGGTCATAGCTGTTTCCTGCAACAAATCC TTCGGGCTTA	*MAT2* knockout PCR primer R1
**B14231**	CAAGGAAGCTTTGGACGACT	*MAT2* knockout PCR primer R2
**B14232**	GCAGCTTACGGTCTGTTTCC	*MAT2* screening primer SO
**B14233**	CGTCCTGGTAGGCAGAGAAC	*MAT2* Southern blot probe primer PO
**B15889**	CGTTCTCTGCCTACCAGGAC	*MAT2* internal primer iLP
**B15890**	CTCTTCCCTCAAACGTCTCG	*MAT2* internal primer iRP
**B11381**	TGAGGGAGAAAAGGTTCC	*MAT2* qRT primer qLP
**B11382**	GGAGGAGGCATTGACTTATTC	*MAT2* qRT primer qRP
**B8097**	CGCCTTCACTGCCATCTTC	*MFα1* qRT primer qLP
**B8098**	ACAAAGGGTCATGCCACCGG	*MFα1* qRT primer qRP

**B9062**	GTTCGAGACTTTCAATGCCC	*ACT1* qRT primer qLP
**B9063**	ACCAGAGTCAAGAACGATAC	*ACT1* qRT primer qRP
**B8517**	CACCCCTTATTTTGCCCTC	*STE12α* qRT primer qLP
**B8518**	GTTTTCGGGTGGGGTGCCA	*STE12α* qRT primer qRP
**B11060**	TTGTGAACGACCACAACCAC	*ZNF2* qRT primer qLP
**B11061**	TGCCTTGCAAGATCACTTTTT	*ZNF2* qRT primer qRP
**B1894**	TTTTACGCTTTTTGCAGATTCCGCCAAA	*MFα1* northern blot primer NP1
**B1895**	GACCACTGTTTCTTTCGTTCT	*MFα1* northern blot primer NP2
**B13320**	ATGGAAGAAGAAGGTACG	*ACT1* northern blot primer NP1
**B13321**	TTAGAAACACTTTCGGTG	*ACT1* northern blot primer NP2
